# The Cohesin Subunit Rad21 Is Required for Synaptonemal Complex Maintenance, but Not Sister Chromatid Cohesion, during Drosophila Female Meiosis

**DOI:** 10.1371/journal.pgen.1004540

**Published:** 2014-08-07

**Authors:** Evelin Urban, Sonal Nagarkar-Jaiswal, Christian F. Lehner, Stefan K. Heidmann

**Affiliations:** 1 Lehrstuhl für Genetik, University of Bayreuth, Bayreuth, Germany; 2 Institute of Molecular Life Sciences (IMLS), University of Zurich, Zurich, Switzerland; Stowers Institute for Medical Research, United States of America

## Abstract

Replicated sister chromatids are held in close association from the time of their synthesis until their separation during the next mitosis. This association is mediated by the ring-shaped cohesin complex that appears to embrace the sister chromatids. Upon proteolytic cleavage of the α-kleisin cohesin subunit at the metaphase-to-anaphase transition by separase, sister chromatids are separated and segregated onto the daughter nuclei. The more complex segregation of chromosomes during meiosis is thought to depend on the replacement of the mitotic α-kleisin cohesin subunit Rad21/Scc1/Mcd1 by the meiotic paralog Rec8. In Drosophila, however, no clear Rec8 homolog has been identified so far. Therefore, we have analyzed the role of the mitotic Drosophila α-kleisin Rad21 during female meiosis. Inactivation of an engineered Rad21 variant by premature, ectopic cleavage during oogenesis results not only in loss of cohesin from meiotic chromatin, but also in precocious disassembly of the synaptonemal complex (SC). We demonstrate that the lateral SC component C(2)M can interact directly with Rad21, potentially explaining why Rad21 is required for SC maintenance. Intriguingly, the experimentally induced premature Rad21 elimination, as well as the expression of a Rad21 variant with destroyed separase consensus cleavage sites, do not interfere with chromosome segregation during meiosis, while successful mitotic divisions are completely prevented. Thus, chromatid cohesion during female meiosis does not depend on Rad21-containing cohesin.

## Author Summary

Meiosis is a specialized form of cell division that ensures production of germ cells with the right number of chromosomes, so that at fertilization the embryo receives complete sets of paternal and maternal chromosomes. The accurate distribution of chromosomes during cell divisions is dependent on a ring-shaped protein complex called cohesin. Cohesin is thought to embrace the chromosomes from the time of their duplication during S-phase until their segregation in the ensuing division. This segregation is facilitated by the controlled proteolytic cleavage of one of the cohesin ring components. Most eukaryotes express specialized variants of this protein: for mitosis the variant Rad21/Scc1/Mcd1 and for meiosis the related protein Rec8. Because Drosophila lacks a clear Rec8 homolog, we have analyzed in the present study whether the mitotic variant Rad21 may also function during meiosis. We have destroyed Rad21-based cohesin by premature cleavage of an engineered Rad21 variant during oogenesis. While we find no indication for effects on the accuracy of meiotic chromosome segregation, Rad21 cleavage results in a premature disassembly of the synaptonemal complex (SC), a structure required for meiotic recombination in Drosophila oocytes. Our interaction studies provide intriguing hints how Rad21 might contribute to SC maintenance.

## Introduction

During meiosis, haploid germ cells are generated from diploid parental cells by two consecutive cell divisions without intervening DNA replication. Before the first meiotic division, homologous chromosomes are paired into bivalents and the two sister centromeres in each homolog are constrained to behave as a functional unit. The two homologous centromeres of each bivalent are bi-oriented in the spindle and segregated apart during the first meiotic division. Thereafter sister centromeres become functionally independent, allowing their bi-orientation and separation during the second meiotic division, very much like during mitosis (for review see: [1]). Importantly, error-free chromosome segregation during each meiotic division (homologs in meiosis I and sisters in meiosis II) does not just depend on regulated centromere behavior but also on temporal and regional control of sister chromatid cohesion.

Sister chromatid cohesion in combination with meiotic crossovers keeps bivalents physically together until the metaphase-to-anaphase transition of the first meiotic division. Crossovers are generated by meiotic recombination between non-sister chromatids of homologous chromosomes. The order of events during initiation of meiotic recombination varies among the organisms. In mice, fungi and plants double strand breaks (DSBs) mark the first event of meiotic recombination, and DSBs are required for the intimate pairing (synapsis) of homologous chromosomes during the extended prophase of meiosis I. In Drosophila, however, synapsis can occur in the absence of prior DSB formation [2]. A unique proteinaceous structure, the synaptonemal complex (SC), is formed during early stages of prophase I between the homologs. SC formation commences with the establishment of the axial elements (AE) which represent a scaffold running alongside the paired sister chromatids within each homolog. Concomitant with pairing of homologs, the AE mature into the lateral elements (LE) of the SC. The LE are connected by perpendicularly oriented transverse filaments (TF) which form the central element (CE) of the SC. In *Drosophila melanogaster*, meiotic recombination only occurs in females and consequently the SC is only assembled during oogenesis. The protein C(2)M has been identified as a component of the LE, and the main element of the TF is the elongated coiled-coil protein C(3)G [3], [4]. Loss of either protein results in a severely compromised SC structure and high levels of chromosome non-disjunction during the meiotic divisions [3], [4]. Proper C(3)G localization requires C(2)M but not *vice versa* [3].

After crossover formation, the SC is disassembled and crossovers mature into chiasmata. Despite SC disassembly, paired homologous chromosomes cannot disjoin, because sister chromatid cohesion distal to the crossover sites prevents terminalization of chiasmata. This cohesion between replicated sister chromatids is mediated by the heterotetrameric ring-shaped cohesin complex (for review see: [5], [6]). Cohesin complex components were originally identified by genetic screens in the yeast *Saccharomyces cerevisiae* [7], [8]. The core cohesin complex consists of the two structural maintenance of chromosomes (SMC) molecules SMC1 and SMC3, which form extended intramolecular coiled-coils and heterodimerize via their hinge regions. An α-kleisin subunit connects SMC1 and SMC3 by binding to their head domains, thus forming a tripartite ring-like structure. The α-kleisin Rad21/Scc1/Mcd1 also recruits the accessory subunit Scc3 (for reviews see: [5], [6]). The cohesin ring most likely embraces the sister chromatids and thereby establishes sister chromatid cohesion topologically [9].

Several eukaryotes are known to express meiosis-specific cohesin components (for review see: [10]). In yeasts the meiosis-specific α-kleisin Rec8 associates with the SMC head domains instead of Rad21/Scc1/Mcd1 [11], [12]. Apart from Rec8 homologs, vertebrate genomes encode yet an additional meiosis-specific α-kleisin, Rad21L, but a direct role for this variant in sister chromatid cohesion awaits demonstration [13], [14], [15]. However, it has been shown in mouse spermatocytes that Rad21L is involved in assembly of the axial elements of the SC [16], [17]. An involvement of cohesin in SC maintenance has been demonstrated previously in several distantly related eukaryotes [11], [18], [19], [20], [21]. Mammalian meiotic cohesin complexes contain the specific subunit SMC1β and the Scc3 homolog STAG3/SA3, while mitotic cells harbor cohesin complexes with SMC1α and either STAG1/SA1 or STAG2/SA2. Not all imaginable combinations of these subunits may be realized in cohesin complexes occurring *in vivo*, but immunoprecipitation of complexes present in mouse testis extracts revealed five variant cohesin complexes with differing subunit composition [15].

Cohesion is abrogated at the metaphase-to-anaphase transition by proteolytic cleavage of the α-kleisin cohesin subunit by the cysteine protease separase, thus opening the cohesin ring and liberating the sister chromatids [22], [23]. In meiosis, two waves of separase activity occur during the two divisions. In meiosis I, separase-dependent cleavage of phosphorylated Rec8, which is present in cohesin complexes located at the chromosome arm regions, allows chiasmata terminalization and hence homolog separation [24], [25], [26], [27]. Importantly, Rec8 in cohesin complexes located within pericentromeric regions is protected from proteolytic cleavage during meiosis I. Proteins of the Shugoshin (Sgo)-family recruit protein phosphatase 2A (PP2A) to the centromeric region, thus keeping Rec8 locally in a cleavage-resistant unphosphorylated state [28], [29]. Consequently, sister centromeres remain paired throughout meiosis I permitting their bi-orientation during meiosis II. A second burst of separase activity destroys pericentromeric cohesion before anaphase II.

In several organisms, the α-kleisin Rad21 is expressed not only before mitotic but also before meiotic divisions. Its role in meiotic sister chromatid cohesion has been discussed controversially. In the mouse, Rad21 is clearly expressed in meiotic cells of both sexes [30]. A number of immunolocalization studies have shown the persistence of Rad21 on mammalian meiotic chromatin at least through meiosis I which has been interpreted as Rad21 possibly serving a cohesive function during meiosis [20], [31], [32], [33], [34]. However, other studies either failed to detect Rad21 in premeiotic S-phase or later stages of rat spermatogenesis [35], or reported Rad21 to apparently leave chromatin before metaphase I in mouse spermatocytes [36]. Elegant functional studies have recently revealed that premature TEV protease-mediated cleavage of all Rad21 has no obvious effect on chromatid cohesion in mouse oocytes, while analogous premature Rec8 cleavage resulted in premature and complete loss of cohesion both in metaphase I, leading to chiasmata resolution, and also in metaphase II [37]. Thus, Rad21 serves no cohesive function during meiosis, at least not in mouse oocytes, and Rec8 cleavage is sufficient for loss of cohesion in both meiotic divisions.

Intriguingly, the Drosophila genome does not contain an obvious Rec8 homolog. However, the SC component C(2)M [3] was shown to be a divergent member of the α-kleisin family by in-depth bioinformatics analyses [38]. Its meiosis-specific expression, its association with the cohesion subunit SMC3, and the high level of chromosome missegregation in *c(2)M* mutants are consistent with C(2)M functioning analogous to Rec8. However, the low level of sister non-disjunction in *c(2)M* mutants, as well as C(2)M localization dynamics during meiosis and the lack of abnormalities after expression of variants predicted to be separase cleavage-resistant, argue against C(2)M being the *bona fide* Rec8 homolog [3], [39]. Two additional genes that seem to be specific to the Drosophila lineage, *solo* and *ord*, qualify to encode meiotic cohesins, as both *solo* and *ord* null mutants show premature dissociation of homologous chromosomes and of sister chromatids, resulting in high frequencies of meiotic non-disjunction events [40], [41], [42]. Also, in *ord* and *solo* mutants, the SC is formed, but it disassembles prematurely [41], [43]. However, neither SOLO nor ORD display similarity with α-kleisins at the primary structure level and there are no reports that either one of the two proteins is a substrate for separase, which is active during the meiotic divisions in Drosophila [39]. Thus, it is at present not clear whether Drosophila harbors, as part of meiotic cohesin complexes, an α-kleisin homolog, which needs to be removed in a separase-dependent manner during the meiotic divisions to allow chromosome/chromatid segregation.

Here we have addressed whether Drosophila Rad21/Verthandi takes over the function of Rec8 by acting as a meiotic α-kleisin and whether it is involved in SC maintenance. We find that experimentally induced premature Rad21 proteolysis during oogenesis does not result in premature chromosome/chromatid separation or chromosome non-disjunction, arguing against an essential contribution of Rad21 to meiotic sister chromatid cohesion. However, maintenance of the SC is clearly dependent on Rad21, which co-localizes with C(2)M and C(3)G in nuclei with a fully formed SC. Moreover, our finding that C(2)M can interact physically with Rad21 allows speculations towards a molecular mechanism for the linkage of the SC to meiotic chromosome cores in Drosophila.

## Results

### Targeted Rad21 inactivation during female meiosis

Rad21 provides essential functions during mitosis. To evaluate whether Rad21 also provides important functions during meiosis, we applied a system allowing controlled Rad21 inactivation specifically during oogenesis. We took advantage of Drosophila strains expressing Rad21 variants that can be proteolytically inactivated by TEV protease. These Rad21 variants contain three consecutive TEV protease cleavage sites at position 271 or 550, as well as a C-terminal myc-epitope tag (Rad21^TEV^-myc). Rad21 mutant rescue experiments have proven these variants to be functional [44]. Furthermore, TEV protease expression has been shown to result in efficient Rad21^TEV^-myc cleavage and consequential inactivation. Cleavage in mutant embryos that rely on Rad21^TEV^-myc as their sole Rad21 species, resulted in completely penetrant premature sister chromatid separation during the first mitosis after onset of TEV protease expression [44].

**Figure 1:**
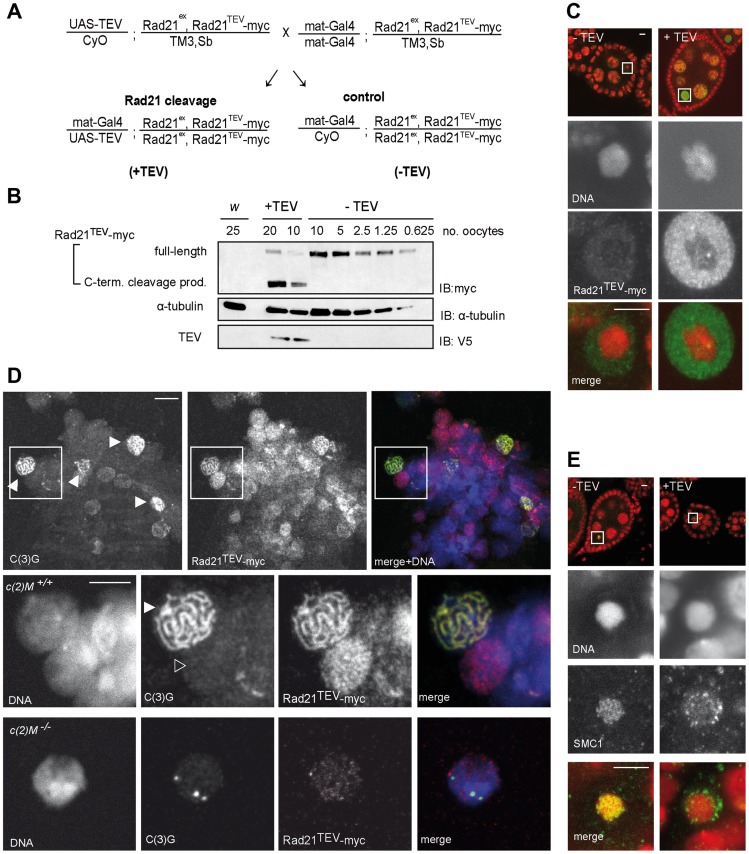
Rad21^TEV^-myc cleavage by TEV protease expression during oogenesis results in cohesin dissociation from chromatin. (A) Crossing scheme illustrating the generation of females, in which the solely expressed Rad21 variant Rad21^TEV^-myc is cleaved during oogenesis due to Gal4 mediated expression of TEV protease (+TEV) as well as of control sibling females (−TEV). *Rad21^ex^*, deletion allele of *Rad21*. (B) Extracts were prepared from stage 14 oocytes obtained from control females (*w^1^*, or −TEV females) or from females expressing TEV protease in the *Rad21^ex^, Rad21^TEV^-myc* homozygous background (+TEV). Proteins were separated by PAGE, blotted and the blot was probed with antibodies against the myc-epitope (top panel), against α-tubulin as loading control (middle panel), and against the V5 epitope to monitor TEV protease expression (bottom panel). The numbers of oocyte equivalents are given on top of the lanes. (C) Immunofluorescence analysis of stage 4–5 egg chambers from Rad21 mutant females (+TEV) or sibling females (−TEV). DNA was stained with Hoechst 33258 and Rad21^TEV^-myc was labeled with anti-myc antibodies. In the upper row, an overview of the egg chambers is presented and the oocyte nucleus is shown enlarged in the other panels. In the merged images, DNA is shown in red and the myc-signal in green. (D) Chromosome spread analysis of germaria from females expressing *Rad21^TEV^-myc*. Within the partially dissociated germarium, some nuclei show the thread-like pattern of C(3)G staining typical for the synaptonemal complex (filled arrowheads in the top panel). In the same nuclei, myc signals are also thread-like and in nuclei of pro-nurse cells, which are negative for C(3)G staining, diffuse myc staining indicates Rad21^TEV^-myc association throughout chromatin (open arrowhead in the enlargements in the bottom panel). In the merged images, DNA is shown in blue, anti-myc in red and C(3)G in green. (E) Immunofluorescence analysis of stage 4–5 egg chambers from Rad21 mutant females (+TEV) or sibling females (−TEV). DNA was stained with Hoechst 33258 and SMC1 with anti-SMC1 antibodies. In the upper row, an overview of the egg chambers is presented and the oocyte nucleus is shown enlarged in the other panels. In the merged images, DNA is shown in red and the SMC1-signal in green. Images are single confocal sections. Exposure times and processing were identical for the images +/− TEV. Scale bars are 5 µm.

To express specifically in the female germline a UAS transgene, which encodes a TEV protease variant with improved catalytic properties (see materials and methods), we used the maternal *alpha-tubulin GAL4*-driver line (*mat-GAL4*). The resulting efficiency of Rad21^TEV^-myc cleavage was assessed with extracts from stage 14 oocytes. Oocytes from sibling females with and without UAS-TEV protease transgene were compared (Fig. 1A, +TEV and −TEV). Immunoblot analyses using antibodies against myc allowed the detection of full-length Rad21^TEV^-myc as well as the C-terminal cleavage product (Fig. 1B). Quantification revealed that around 95% of Rad21^TEV^-myc was cleaved in the TEV protease expressing oocytes (Fig. 1B).

To assess phenotypic consequences of Rad21^TEV^-myc cleavage *in situ*, we immunolabeled ovarioles with anti-myc antibodies. In the absence of TEV protease expression, only weak signals were obtained within the nucleoplasm surrounding the karyosome, the highly condensed chromatin of the oocytes (Fig. 1C, −TEV). However, upon expression of TEV protease, strong anti-myc signals were detected in the nucleoplasm (Fig. 1C, +TEV), indicative of Rad21^TEV^-myc cleavage product accumulation. The inability to detect uncleaved Rad21^TEV^-myc localizing on oocyte chromatin in these whole mount preparations could, in principle, be due to accessibility problems. Thus, we also stained chromosome spread preparations of germaria and early egg chambers. While this method did not allow the unambiguous assignment of nuclei to specific stages of oogenesis, we clearly detected nuclear anti-myc signals in cells of germaria. Most importantly, while the signals were diffuse in nuclei without an SC, we obtained strong anti-myc signals co-localizing with the synaptonemal complex (SC) component C(3)G in the typical thread-like pattern in pro-oocytes (Fig. 1D). To confirm the presence of Rad21 in the SC, we also analyzed spread preparations of ovarioles from females expressing a functional Rad21-EGFP variant by double labeling with anti-EGFP and anti-C(3)G antibodies. We again observed co-localization in nuclei with a fully formed SC, corroborating our results obtained for localization of Rad21^TEV^-myc.

Rad21 is, together with SMC1 and SMC3, part of the tripartite cohesin ring, embracing sister chromatids after DNA replication (for review see [5]). As the cohesin rings are bound to chromatin in a topological fashion, SMC1 and SMC3 are expected to dissociate from chromatin upon Rad21^TEV^-myc cleavage. In contrast to Rad21, SMC1 and SMC3 can be readily visualized on meiotic chromatin by immunostaining of Drosophila ovariole whole mount preparations [45]. There, SMC1 and SMC3 are associated with the lateral elements of the SC [45]. Indeed, while a characteristic pattern of SMC1 can be detected in the karyosome in the absence of TEV protease expression, SMC1 is delocalized upon Rad21^TEV^-myc cleavage (Fig. 1E). We conclude that in our system Rad21^TEV^-myc is efficiently cleaved during oogenesis, and that this cleavage leads to premature dissociation of cohesin from meiotic chromatin.

### Premature Rad21 cleavage causes precocious disassembly of the synaptonemal complex

**Figure 2:**
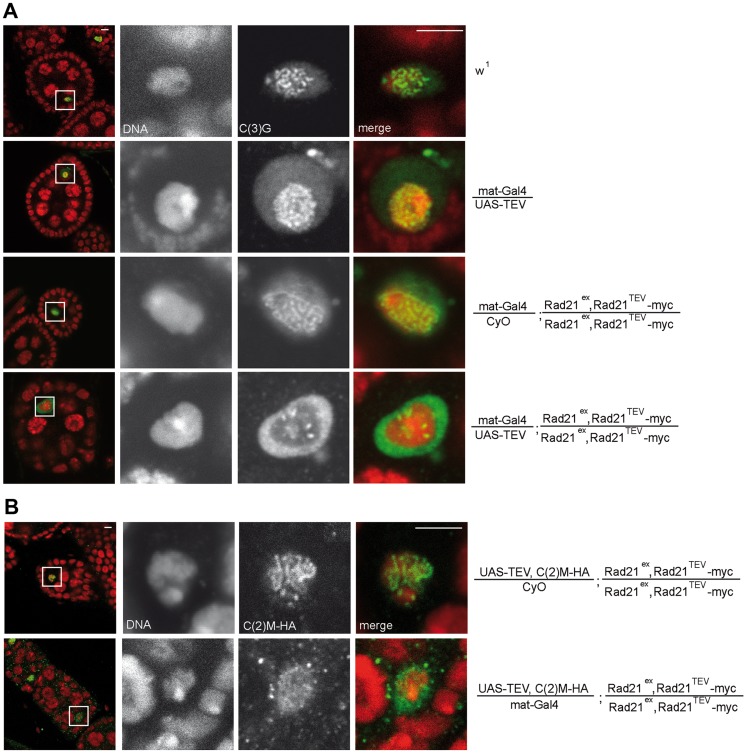
Premature Rad21^TEV^-myc cleavage during oogenesis results in precocious SC disassembly. (A) Immunofluorescence analysis of stage 4–5 egg chambers from wild type females (w^1^), females with *GAL4*-driven expression of TEV protease in a *Rad21* wild type background (mat-Gal4/UAS-TEV), females expressing only *GAL4* in a *Rad21^TEV^-myc* rescue background (mat-Gal4/CyO; Rad21^ex^, Rad21^TEV^-myc/Rad21^ex^, Rad21^TEV^-myc), or females with *GAL4*-driven expression of TEV protease in a *Rad21^TEV^-myc* rescue background (mat-Gal4/UAS-TEV; Rad21^ex^, Rad21^TEV^-myc/Rad21^ex^, Rad21^TEV^-myc). DNA was stained with Hoechst 33258 and C(3)G was labeled with anti-C(3)G antibodies. In the left column, an overview of the egg chambers is presented and the oocyte nucleus is shown enlarged in the other panels. In the merged images, DNA is shown in red and the C(3)G-signal in green. Note the enrichment of C(3)G signal in the nucleoplasm after TEV-mediated Rad21^TEV^-myc cleavage (bottom panels). (B) Egg chambers from females expressing *C(2)M-HA* under control of the *c(2)M* genomic regulatory sequences in a *Rad21* mutant background (UAS-TEV, C(2)M-HA/mat-Gal4; Rad21^ex^, Rad21^TEV^-myc/Rad21^ex^, Rad21^TEV^-myc) or from sibling females not expressing TEV protease (UAS-TEV, C(2)M-HA/CyO; Rad21^ex^, Rad21^TEV^-myc/Rad21^ex^, Rad21^TEV^-myc) were analyzed by immunolabelling with anti-HA antibodies. Images are single confocal sections. Exposure times and processing were identical for the images +/− TEV. Scale bars are 5 µm.

**Figure 3:**
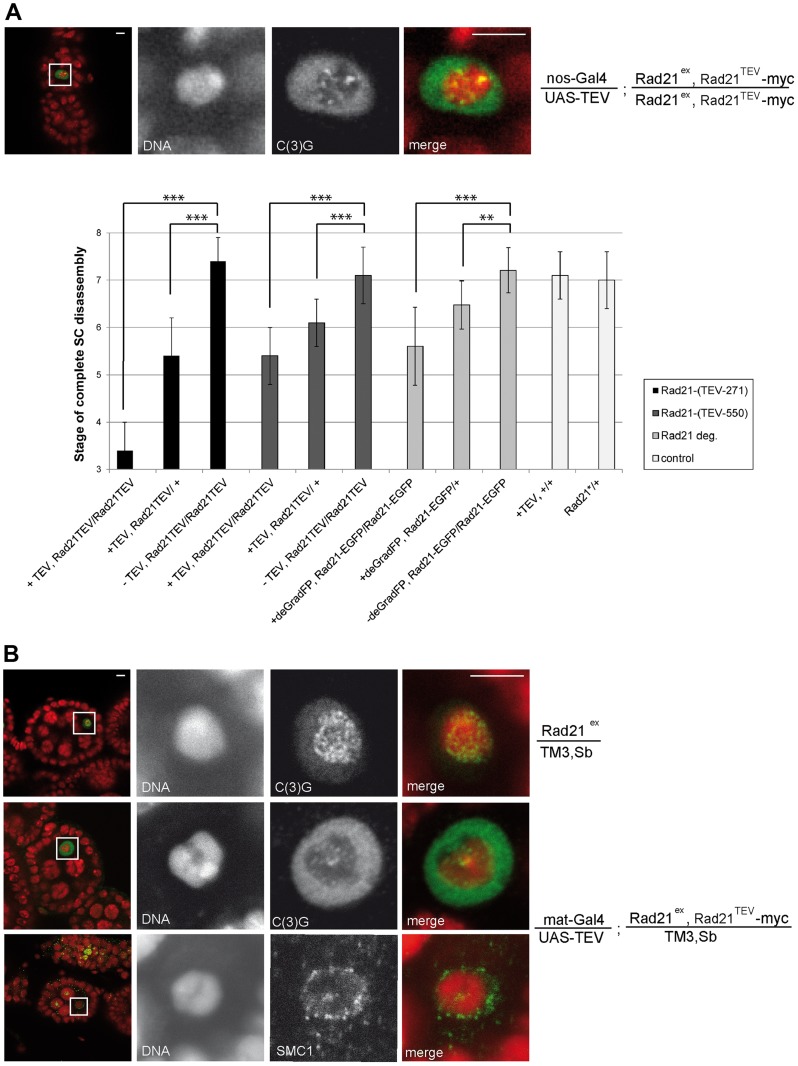
Premature SC disassembly can be triggered by Rad21 removal using different driver/transgene combinations. (A) Immunofluorescence analysis of a stage 4–5 egg chamber from a female expressing TEV protease driven by *nos-GAL4* in a *Rad21^TEV^-myc* rescue background. DNA was stained with Hoechst 33258 and C(3)G was labeled with anti-C(3)G antibodies. In the merged images, DNA is shown in red and the C(3)G-signal in green. The quantification illustrates the mean stage of SC disassembly in ovarioles of females with the indicated genotype. +TEV, TEV protease expression driven by *nos-GAL4*; −TEV, sibling controls not expressing TEV protease; Rad21TEV, indicates presence of the recombinant chromosome *Rad21^ex^, Rad21^TEV^-myc*. Rad21-EGFP, indicates presence of the recombinant chromosome *Rad21^ex^, Rad21-EGFP*; +deGradFP; NSlmb-vhhGFP4 expression driven by *nos-GAL4*; -deGradFP, sibling controls not expressing *NSlmb-vhhGFP4*. Black bars, TEV cleavage site position at aa 271 of Rad21; dark gray bars, TEV cleavage site position at aa 550 of Rad21; light grey bars, presence of the recombinant chromosome *Rad21^ex^, Rad21-EGFP*; white bars, controls expressing TEV protease in a wild type background (+TEV, +/+) or *Rad21^ex3^* heterozygous females not expressing any transgene (Rad21*/+). In each case, 33 to 34 ovarioles were scored, except for +deGradFP, Rad21-EGFP/+ (21 ovarioles). Error bars represent standard error. ***: p<0.0001; **: p = 0.0002; as determined by pairwise comparisons using the Mann-Whitney U-test. (B) Immunofluorescence analysis of stage 4–5 egg chambers from females expressing TEV protease driven by *mat-GAL4* in a *Rad21^ex^, Rad21^TEV^-myc* heterozygous background (mat-Gal4/UAS-TEV; Rad21^ex^, Rad21^TEV^-myc/TM3, Sb) or control females heterozygous for the *Rad21* excision allele (Rad21^ex^/TM3, Sb). DNA was stained with Hoechst 33258 and C(3)G or SMC1 were labelled with specific antibodies. In the left column, an overview of the egg chambers is presented and the oocyte nucleus is shown enlarged in the other panels. In the merged images, DNA is shown in red and the C(3)G-signal/SMC1-signal in green. Note that even in the presence of one wild type *Rad21* allele, cohesin leaves chromatin and the SC disassembles prematurely after forced Rad21 cleavage. Scale bars are 5 µm.

Since Rad21^TEV^-myc cleavage occurs during a developmental stage when the SC is fully formed in the oocyte nucleus (TEV protease expression driven by *mat-GAL4* can be detected starting in region 3 of the germaria; Fig. S1A), we addressed possible phenotypic consequences on SC integrity. Immunolabeling of the SC-components C(3)G and an HA tagged variant of C(2)M within wild type oocyte nuclei of stage 4–5 egg chambers resulted in the expected ribbon-like SC staining (Fig. 2) [3], [4]. TEV protease expression in a background without Rad21^TEV^-myc but with wild type Rad21 did not affect the SC-associated anti-C(3)G signals that were just like in wild type ovarioles (Fig. 2A). A normal C(3)G staining pattern was also observed in control ovarioles expressing Rad21^TEV^-myc in the *Rad21* mutant background in the absence of TEV protease (Fig. 2A), and in ovarioles from Rad21^+^/Rad21^−^ heterozygote individuals (Fig. 3B). However, the characteristic ribbon-like C(3)G staining was almost completely lost from the oocyte chromatin after TEV protease expression in a background with exclusively Rad21^TEV^-myc, and C(3)G accumulated instead in the nucleoplasm (Fig. 2A). We point out that in these same ovarioles at earlier stages within the germarium, C(3)G staining still revealed the normal ribbon-like structures (Fig. S2), as expected, because mat-GAL4 driven TEV protease expression is not yet detectable at these early stages (Fig. S1A).

To assess whether the localization of the lateral SC component C(2)M is also affected after premature Rad21 cleavage, we generated flies, which express *c(2)M-HA* under genomic control in a *rad21* mutant background rescued by *Rad21^TEV^-myc* expression. When TEV protease was expressed in these ovarioles, the ribbon-like C(2)M-HA-staining typical for the SC also disappeared from the meiotic chromatin and C(2)M-HA distributed throughout the nucleus (Fig. 2B).

Thus, our results suggest that the SC disassembles as a consequence of Rad21^TEV^-myc cleavage. To evaluate whether the observed phenotype is due to a dominant negative effect of the particular cleavage products generated, we analyzed the dependence of the phenotype on the precise position of the TEV cleavage sites within Rad21^TEV^-myc. Moreover, to rule out effects of the GAL4 driver background, we repeated the experiments with a different driver, *nanos (nos)-GAL4*. *nos-GAL4* expression commences earlier during oogenesis, in region 2a of the germarium (Fig. S1B). Indeed, TEV protease expression directed by this driver resulted in premature SC disassembly at an even earlier stage during oogenesis (*nos-GAL4*: stage 3.4+/−0.6 (n = 34); *mat-GAL4*: stage 5.3+/−0.6 (n = 30); P<0.0001; Mann-Whitney U-test; Fig. 3A). Moreover, SC disassembly was observed to occur at an earlier stage with Rad21^TEV^-myc having the TEV cleavage sites after amino acid 271 compared to after amino acid 550 (position 271: SC disassembly + TEV at stage 3.4+/−0.6 vs. − TEV at stage 7.4+/−0.5; position 550: SC disassembly + TEV at stage 5.4+/−0.6 vs. −TEV stage 7.1+/−0.6; Fig. 3A).

Although *nos-GAL4* driven TEV protease expression can be detected early in region 2a of the germarium, establishment of the SC was not affected (Fig. S2). Analysis of spread preparations of germaria revealed clear evidence of initial Rad21 degradation before SC disassembly (Fig. S3). Therefore, the SC disassembly which is observed during later oogenesis might depend on complete Rad21 degradation.

Taken together, our results demonstrate that precocious SC disassembly is a robust phenotype that is observed with different *GAL4* drivers and different TEV cleavage site insertion positions within Rad21^TEV^-myc.

Interestingly, premature SMC1 delocalization and SC disassembly also occurred when Rad21^TEV^-myc was cleaved by TEV protease expression in the presence of one wild type *Rad21^+^* allele (Fig. 3). While in these cases the SC stayed intact longer than in the *Rad21* mutant situation, the difference in SC disassembly timing compared to the control situation was still highly significant (position 271: stage 5.4+/−0.8 vs. stage 7.4+/−0.5; position 550: stage 6.1+/−0.5 vs. stage 7.1+/−0.6; Fig. 3A). The precocious SC disassembly is not due to the reduced *Rad21^+^* gene dosage, because the dynamics of SC disassembly is like wild type in females heterozygous for *Rad21^ex^* without any ectopic Rad21^TEV^-myc cleavage (Fig. 3). Moreover, we re-iterate that TEV protease expression in a background with wild type Rad21 has no effect when Rad21^TEV^-myc is not expressed (Fig. 2A and 3A).

As an independent approach to remove Rad21 from developing egg chambers, we applied targeted destruction of GFP-tagged proteins by the deGradFP system [46]. In this system, GFP-fused proteins are recruited to a recombinant SCF ubiquitin ligase generated by expression of a specific single-chain anti-GFP antibody fused to the F-box region of Slmb (NSlmb-vhhGFP4). The recruitment of GFP fusions by NSlmb-vhhGFP4 results in their proteasomal degradation. We constructed strains in which *Rad21* mutants are rescued by the expression of *Rad21-EGFP*. NSlmb-vhhGFP4 expression driven by *mat-GAL4* markedly reduced Rad21-EGFP protein levels (Fig. S4). *nanos-GAL4* driven expression of the NSlmb-vhhGFP4 fusion protein again resulted in premature dissociation of the SC. The difference in SC disassembly timing compared to the control situation (*Rad21^ex^, Rad21-EGFP* homozygous females without *NSlmb-vhhGFP4* expression) was again highly significant (*Rad21^ex^, Rad21-EGFP* homozygous females + *NSlmb-vhhGFP4*: stage 5.6+/−0.8 vs. stage 7.2+/−0.5; *Rad21^ex^, Rad21-EGFP* heterozygous females + *NSlmb-vhhGFP4*: stage 6.5+/−0.5 vs. stage 7.1+/−0.5; Fig. 3A). This premature SC disassembly after proteasomal degradation of Rad21-EGFP confirms that loss of Rad21 results in SC disintegration. This SC instability therefore does not depend on the presence of Rad21^TEV^-myc cleavage fragments, which are generated after TEV protease expression, and which might in principle have a dominant effect.

Although the majority of the SC components C(3)G and C(2)M leaves the oocyte chromatin after forced Rad21 cleavage, some bright staining patches remain (Fig. 2A, Fig. 3A). As residual SC components have been described to remain associated with clustered centromeres after normal SC disassembly [47], we analyzed whether residual C(3)G after ectopic Rad21^TEV^-myc cleavage was colocalized with centromeres. Upon premature Rad21 cleavage, we indeed found colocalization of the centromeric H3 variant Cid/Cenp-A with persisting C(3)G patches (Fig. S5), suggesting that association of the SC with centromeric regions might not depend on Rad21-containing cohesin. Taken together, our data imply that the integrity of the mitotic α-kleisin cohesin subunit Rad21 is required for SC maintenance at chromosome arms during *Drosophila* oogenesis.

### Rad21 binds to the N terminus of the SC component C(2)M

**Figure 4:**
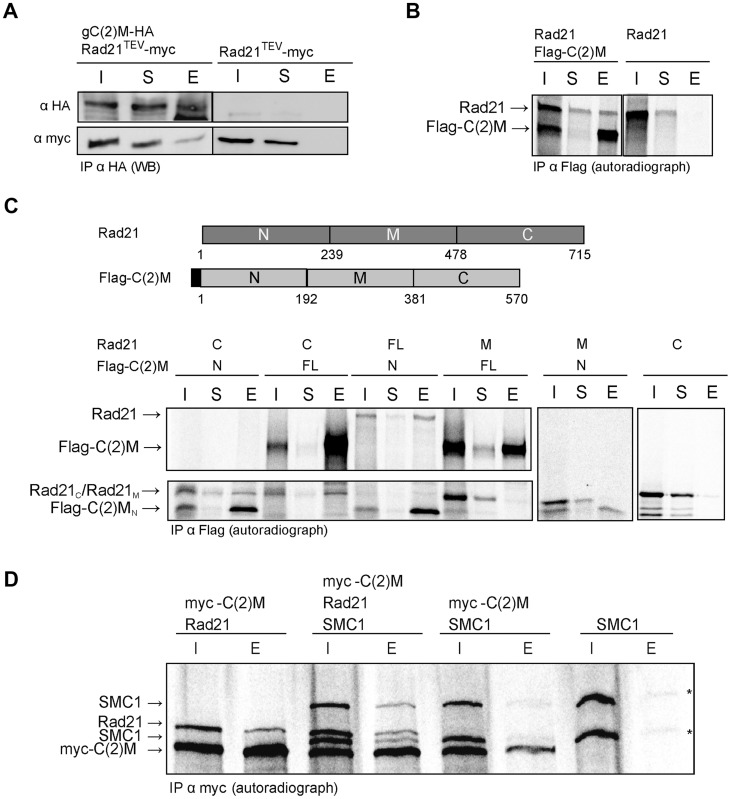
C(2)M physically interacts with Rad21. (A) Extracts from 0–1.5 h old embryos expressing either h old embryos expressing either *gC(2)M-HA* together with *Rad21^TEV^-myc* or just *Rad21^TEV^-myc* were subjected to immunoprecipitation (IP) with mouse anti-HA antibodies. Bound proteins were eluted (E), separated by SDS-PAGE together with input (I) and supernatant after IP (S) samples, and analyzed by western blotting (WB). The blotted samples were probed with anti-HA antibodies to control for immunoprecipitation efficiency and anti-myc antibodies to assess co-precipitation of Rad21^TEV^-myc. The samples were run on the same gel but not immediately adjacent to each other. Lanes removed from the image are indicated by the vertical black line (B) Full length versions of Rad21 and Flag-epitope tagged C(2)M were synthesized by coupled *in vitro* transcription/translation (IVT) in the presence of [^35^S]methionine. IVT reactions were subjected to immunoprecipitation using anti-Flag antibodies. Radioactively labelled proteins were detected by autoradiography. The samples were run on the same gel but not immediately adjacent to each other. Lanes removed from the image are indicated by the vertical black line (C) Schematic of the various Rad21 and Flag-C(2)M fragments assayed for interaction in the coupled IVT-IP experiments. Rad21 fragments were untagged, while all C(2)M fragments were N-terminally fused to 3 copies of the Flag epitope. The proteins were either of full length (FL) or represented the N-terminal part (N), the middle part (M) or the C-terminal part (C) of Rad21 or C(2)M. After IVT-IP using anti-Flag antibodies the samples (I, input; S, supernatant, E, eluate) were separated by SDS-PAGE and radioactively labelled proteins were detected by autoradiography. The migration position of the various fragments is indicated on the left. (D) Coupled IVT-IP of full-length versions of SMC1, Rad21, and myc-C(2)M. After IP using anti-myc antibodies, input (I) and eluate (E) fractions were analyzed. Note that IVT of SMC1 resulted in two protein species, as indicated by asterisks on the right.

To address how Rad21 interacts with the SC, we first analyzed whether Rad21 protein might bind to the SC component C(2)M. We performed co-immunoprecipitation experiments using protein extracts prepared from fly embryos expressing *c(2)M-HA* and *Rad21^TEV^-myc*. The presence of C(2)M and Rad21 in early embryos has been demonstrated previously [39]. Indeed, Rad21^TEV^-myc was co-precipitated with C(2)M-HA. In control experiments, where we used the same anti-HA antibodies for immunoprecipitation from an extract containing Rad21^TEV^-myc, but not C(2)M-HA, we were unable to pull down Rad21^TEV^-myc (Fig. 4A), ruling out a non-specific association of Rad21^TEV^-myc with HA-antibodies or beads. To obtain independent support for an interaction between Rad21 und C(2)M, we conducted *in vitro* pull-down assays. To this end, we used an *in vitro* transcription/translation (IVT) system to synthesize Rad21 and Flag epitope-tagged C(2)M in a reticulocyte lysate in the presence of [^35^S]methionine. Autoradiography of the samples after anti-Flag immunoprecipitation revealed that Rad21 specifically bound to Flag-C(2)M (Fig. 4B). To delineate the interacting domains of the two proteins, we repeated the assay with *in vitro* synthesized fragments of both proteins. These experiments revealed that an N-terminal fragment of C(2)M (C(2)M_N_, aa 1–191) is sufficient to precipitate Rad21 (Fig. 4C). Furthermore, the C-terminus of Rad21 (Rad21_C_, aa 478–715) is sufficient for interaction with Flag-C(2)M or Flag-C(2)M_N_ (Fig. 4C). None of the other fragments were able to mediate an interaction in this system (Fig. 4C). In the same assay system, we neither detected an interaction between Rad21 and one of the other SC components, C(3)G or Corona, nor an interaction between Rad21 and the cohesion proteins ORD or SOLO (data not shown). C(2)M has been found in a complex with SMC3 [39]. If C(2)M binds directly to the SMC heads, as it is regarded typical for α-kleisins, one would expect the binding of C(2)M and Rad21 to the SMC cohesin subunits to be mutually exclusive. Using the IVT system, we analyzed the binding potential of C(2)M and Rad21 towards SMC1. In these experiments, C(2)M was N-terminally fused with a 6× myc epitope tag, co-expressed with Rad21 and/or SMC1 and anti-myc immunoprecipitates were analyzed (Fig. 4D). While Rad21 could be readily co-immunoprecipitated together with myc-C(2)M, co-immunoprecipitation of SMC1 depended on the presence of Rad21. Thus, these data suggest that myc-C(2)M does not bind directly to SMC1 but that Rad21 mediates the association of C(2)M with SMC1. Taken together, our immunoprecipitation analyses reveal a novel interaction between the two α-kleisin proteins Rad21 and C(2)M. Specifically, we show that the C-terminus of Rad21 binds to the N-terminus of C(2)M, suggesting that this interaction mediates the association of C(2)M with the core cohesin complex. *In vivo*, localization of Rad21 and C(2)M are mutually dependent, consistent with an interaction of these two proteins (Figs. 1D and 2B).

### Rad21 cleavage does not profoundly affect cohesion during the meiotic divisions

**Figure 5:**
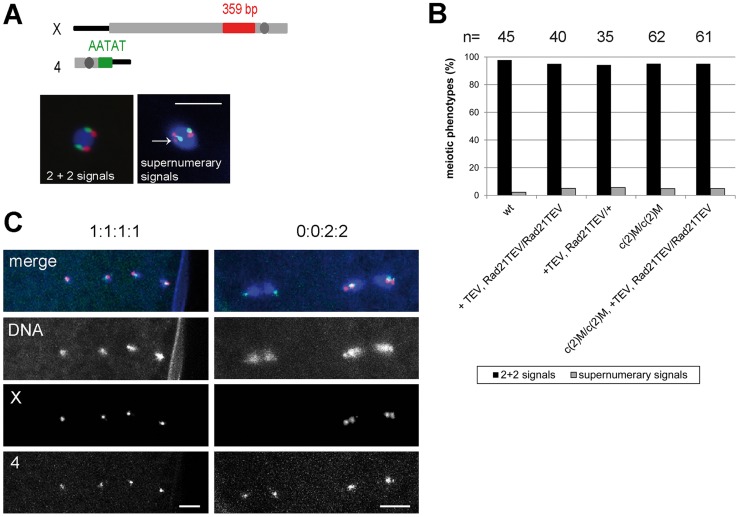
Ectopic Rad21 cleavage does not result in metaphase I alignment defects. (A) Schematic illustrating the FISH probes used to detect the X and 4^th^ chromosomes in late stage oocyte nuclei. Centromeres are indicated by dark grey circles. The X chromosome-specific 359 bp probe was labelled with Alexa 647 and the 4 bp probe was labelled with Alexa 647 and the 4^th^ chromosome specific AATAT probe with Alexa 555. The images on the bottom show examples for the two different categories defined to score the FISH phenotype. The arrow indicates a supernumerary signal for the X chromosome-specific probe. Scale bar is 5 µm. (B) Quantification of the phenotypes of late stage oocyte nuclei after FISH using the X and 4^th^ chromosome-specific probes. The females used to prepare the oocytes had the genotypes *w^1^* (wt), or *mat-GAL4/UAS-TEV; Rad21^ex^, Rad21^TEV^-myc/Rad21^ex^, Rad21^TEV^-myc* (+TEV, Rad21TEV/Rad21TEV) or *mat-GAL4/UAS-TEV; Rad21^ex^, Rad21^TEV^-myc/TM3, Sb* (+TEV, Rad21TEV/+) or *c(2)M^EP2115^/c(2)M^EP2115^* (c(2)M/c(2)M) or *c(2)M^EP2115^, mat-GAL4/c(2)M^EP2115^, UAS-TEV; Rad21^ex^, Rad21^TEV^-myc/Rad21^ex^, Rad21^TEV^-myc* (c(2)M/c(2)M, +TEV, Rad21TEV/Rad21TEV). The total numbers of oocytes scored are given on top of the diagram. (C) FISH analysis of anaphase II figures with probes detecting the X-chromosome (red in the merged images) and the 4^th^ chromosome (green in the merged images) in eggs laid by females with the genotype *mat-GAL4/UAS-TEV; Rad21^ex^, Rad21^TEV^-myc/Rad21^ex^, Rad21^TEV^-myc*. In 82/83 cases, a normal 1∶1∶1∶1 distribution was observed for both probes (left panels). In 1/83 cases, a 0∶0∶2∶2 distribution for the X-chromosome was recorded, indicative of non-disjunction in meiosis I (right panels).

Having established that Rad21^TEV^-myc cleavage results in premature SC disassembly, we wondered whether additional late meiotic processes were affected. If Rad21 is required for cohesion between sister chromatids during the meiotic divisions, one would expect precocious separation of sister chromatids in the Rad21 mutant situation, and consequently chromosome missegregation. Classical genetic non-disjunction assays are not possible in our system, because TEV protease expression in our experiments inactivates the essential maternal Rad21 contribution and therefore results in complete female sterility. In multiple experiments, after *mat-GAL4* driven TEV protease expression causing Rad21^TEV^-myc cleavage, no larvae hatched from the eggs laid by those females. Immunofluorescence analysis of these embryos revealed massive defects already during the very early zygotic divisions. Fragmented and unequally sized DNA masses could be observed, organizing multiple and/or multipolar spindles (Fig. S6). Most embryos appeared to have arrested in a metaphase-like state. Thus, the sterility of these females precluded scoring of genetic markers in adult progeny. As an alternative approach, we applied fluorescent *in situ* hybridization (FISH) to detect chromosome-specific regions in metaphase I-arrested oocytes and analyzed them with reference to precocious chromosome separation. We used an X-chromosome-specific probe (359 bp repeat) and a chromosome 4-specific probe (AATAT)_6_ (Fig. 5A). The observed phenotypes were assigned to two different categories: (1) normal meiotic figures exhibiting 2+2 FISH signals, and (2) precocious separation of chromatids as indicated by supernumerary FISH signals (>2 FISH signals for at least one of the probes; Fig. 5A). In the wild type situation, 98% of the oocytes showed normal meiotic figures, as indicated by the two signals for the different chromosomes (Fig. 5A and B). 2% of the wild type oocytes contained more than 2 signals for one of the two probes (n = 45). After TEV protease-mediated Rad21^TEV^-myc cleavage, the distribution of phenotypes was similar: 95% of the oocytes displayed a normal arrangement, and 5% of the analyzed oocytes had an elevated number of FISH signals indicative of premature chromatid separation (n = 40). Also, oocytes from *c(2)M* mutant females which have been shown previously to display high levels of meiosis I non-disjunction [3], [39], showed no increase of premature chromatid separation (5% of the *c(2)M* mutant oocytes exhibited supernumerary FISH signals (n = 62). Finally, we analyzed oocytes of individuals in which Rad21^TEV^-myc cleavage was performed in the *c(2)M* mutant background (Fig. 5B). In this constellation, again, only 5% of the oocytes were assigned to the ‘supernumerary FISH signals’ category (n = 61). On the contrary, very similar analyses in *ord* mutants using a probe directed against the same repetitive region of the X-chromosome revealed a high proportion (46%) of prometaphase oocyte nuclei with three or four FISH signals, indicating loss of cohesion [48]. Thus, our data do not suggest any additional contribution of Rad21 to chromatid cohesion at this developmental stage during oogenesis. To analyze meiotic divisions directly, we also performed FISH on very early embryos shortly after egg deposition. In this experiment, we observed in the majority of cases a correct 1∶1∶1∶1 distribution of FISH signals among the four meiosis II products in the Rad21 mutant situation, indicative of normal segregation in both meiotic divisions. In only one out of 83 cases, we detected a clear example of missegregation (in meiosis I) with a signal distribution of 0∶0∶2∶2 (Fig. 5C). Taken together, after efficient cleavage of Rad21^TEV^-myc in the oocytes, the effects on meiotic chromosome segregation, if any, were very mild. These findings indicate that Rad21 is not required for sister chromatid cohesion in the oocyte nuclei, in contrast to ORD and SOLO [40], [41].

ORD and/or SOLO may function to maintain sister chromatid cohesion and thereby explain that Rad21 is not required during the meiotic divisions for normal chromosome segregation. To evaluate this possibility, we analyzed the localization of a functional Venus-SOLO variant [42]. In wild-type egg chambers, Venus-SOLO is localized in the vicinity of centromere clusters [41]. Upon Rad21^TEV^-myc cleavage, Venus-SOLO persisted in a dot-like pattern co-localizing with Cid/Cenp-A and C(3)G remnants (Fig. S7). In contrast, the ribbon-like C(3)G staining characteristically present in early egg chambers in wild type [4] (Fig. 2) was largely dissipated in the oocyte nucleoplasm, confirming that premature SC disassembly after Rad21^TEV^-myc cleavage occurred also in the Venus-SOLO background as expected (Fig. S7). The observed pericentromeric presence of SOLO even after Rad21^TEV^-myc cleavage is consistent with the notion that SOLO might render Rad21 dispensable during the meiotic divisions.

**Figure 6:**
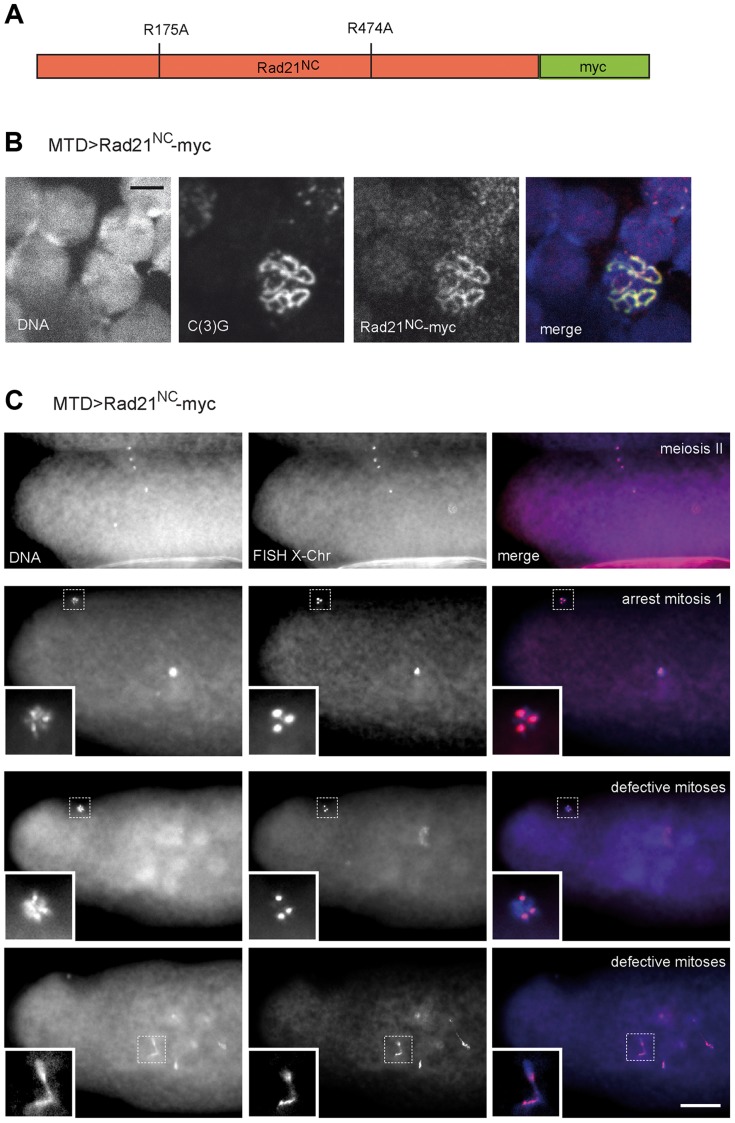
Expression of Rad21 with mutated separase cleavage sites does not impair meiotic divisions. (A) Schematic illustration of the Rad21 variant with mutated separase cleavage sites (Rad21^NC^-myc). The arginines within the separase consensus sites at positions 175 and 474 were changed to alanines. (B) Chromosome spread analysis of germaria from females expressing *Rad21^NC^-myc* under control of *MTD-GAL4*. The non-cleavable Rad21 variant co-localizes with the synaptonemal complex component C(3)G indicating the incorporation of Rad21^NC^-myc into meiotic chromatin. In the merged image, DNA is shown in blue, the myc-signal in red, and the C(3)G-signal in green. Scale bar is 5 µm. (C) Embryos from mothers expressing *Rad21^NC^-myc* under control of *MTD-GAL4* showed normal meiosis II figures (upper row). Each of the four meiotic products contains one X-chromosome-specific FISH signal. During later stages, three meiotic products collapse into the polar body, containing three X-chromosome-specific FISH signals (second row; inset). The zygotic nucleus appears hypercondensed. In the rare cases where multiple DNA masses were apparent within the embryo, they frequently exhibited pronounced anaphase bridges (defective mitoses; last row). Despite these defects the polar bodies have normal appearance and exhibit three X-chromosome-specific FISH signals (second to last row). The images in the bottom two rows represent different focal planes of the same embryo. In the merged images, DNA is shown in blue and the FISH signal in red. Scale bar is 50 µm.

Alternatively, the apparently normal meiotic chromosome segregation observed after TEV protease-mediated Rad21^TEV^-myc cleavage before the meiotic divisions might also reflect the presence of a low, but sufficient, amount of residual non-cleaved Rad21^TEV^-myc. If sister chromatid cohesion during the meiotic division was indeed provided by Rad21 containing cohesin, separase-mediated Rad21 cleavage would be predicted to be essential for normal chromosome segregation during meiosis. To evaluate the significance of separase-mediated Rad21 cleavage during meiosis, we expressed a variant of Rad21-myc, in which the predicted separase cleavage sites (EXXR at positions 172–175 and 471–474) were destroyed by exchange of the arginines with alanines (Fig. 6A). This variant, dubbed Rad21^NC^-myc (non-cleavable), is predicted to be highly toxic in mitotically proliferating cells. Indeed, after expression of Rad21^NC^-myc in the proliferating eye imaginal disc, adults with severely reduced eyes were obtained (Fig. S8). After expression during oogenesis, Rad21^NC^-myc was observed to be co-localized with C(3)G in the SC during the early stages (Fig. 6B), indicating that this mutant is still capable to associate with chromatin. However, those females expressing Rad21^NC^-myc during oogenesis were almost completely sterile. Importantly, abnormalities were only apparent after normal completion of meiosis. All the late meiotic figures observed in early embryonic progeny were normal (13 clear MII anaphase/telophase figures among 230 analyzed embryos; Fig. 6C). FISH analysis demonstrated that X-chromosome segregation during meiosis is not perturbed by Rad21^NC^-myc expression (Fig. 6C). Apart from late meiotic figures, also all of the remnants of the polar bodies displayed a normal morphology and the expected three X chromosome FISH signals. In contrast to the meiotic divisions, however, mitotic divisions during early embryogenesis were severely affected by the maternally expressed Rad21^NC^-myc. In many embryos, strong defects were apparent already during mitosis 1, as only a single DNA mass was observed in the interior of the embryos (Fig. 6C). During this as well as later mitoses, prominent anaphase bridges were detected and X chromosome FISH revealed chromosome stretching (Fig. 6C), as expected after expression of a Rad21 variant that can no longer be cleaved by separase to initiate a normal anaphase. The observed drastic effect of Rad21^NC^-myc on mitotic, but not meiotic, chromosome segregation further confirms that Rad21 is not functioning as an essential α-kleisin component of meiotic cohesin.

## Discussion

Rad21/Scc1 has been established as the α-kleisin subunit of cohesin in mitotic cycles from yeast to man. Even though Rad21/Scc1 is expressed during meiosis, a cohesive role in the meiotic divisions has been ruled out for murine female meiosis [37]. In *Saccharomyces cerevisiae*, Scc1 levels decline sharply when cells enter meiosis, while Rec8 abundance increases dramatically [11]. *scc1* mutants have mild meiotic phenotypes and separase-dependent Rec8 cleavage is required for meiotic chromosome segregation [11], [26]. Likewise in *Schizosaccharomyces pombe*, Rec8, but not Rad21, localizes throughout chromatin during prophase of meiosis I and Rec8 cleavage is required for both meiosis I and meiosis II chromosome segregation [12], [27]. Thus, the emerging view is that during entry into the meiotic program a switch occurs from Rad21 containing cohesin complexes to Rec8 containing cohesin complexes, which are responsible for establishing and maintaining sister chromatid cohesion throughout meiosis. This initially simple picture has become more complicated in vertebrates with the discovery of Rad21L, whose possible function in sister chromatid cohesion remains to be addressed [13], [14], [15], [17]. The situation is even more puzzling in Drosophila, because an unambiguous Rec8 homolog appears to be missing and unrelated proteins like ORD and SOLO with no obvious homology to α-kleisins functionally qualify as cohesion proteins. Originally, C(2)M was assigned as the Drosophila Rec8 homolog based on its meiotic expression profile and its membership in the α-kleisin protein family [38]. However, we have shown that C(2)M accumulates on chromatin only after completion of premeiotic S-phase, appears to dissociate long before pro-metaphase I, and that the mutation of putative separase cleavage sites had no effect on C(2)M function, which is inconsistent with a behavior expected for a meiotic cohesin component [39]. Moreover, *c(2)M* mutants display high levels of non-disjunction only in meiosis I, and not in meiosis II, and SMC1/SMC3 is able to localize to meiotic chromatin in the absence of C(2)M [3], [45]. Thus, it remains an open question whether Drosophila expresses a meiotic α-kleisin, which needs to be removed in a stepwise fashion during the two meiotic divisions. In the present study, we have investigated whether Rad21 might function also as a meiotic kleisin in Drosophila, in addition to its established role as mitotic cohesin subunit. As a precedent, a recent study has shown that the protist *Tetrahymena thermophila* uses only one α-kleisin both in mitosis and meiosis [49].

If Rad21 fulfilled a cohesive function during the meiotic divisions in Drosophila, one would expect to observe after ectopic Rad21 cleavage a dissociation of paired homologous chromosomes and premature sister chromatid separation during the extended pachytene stage of meiosis I and, in addition, missegregation of chromosomes in both meiotic divisions. We have performed immunostainings against the constitutive Drosophila kinetochore component Cenp-C and we did not notice an elevated number of Cenp-C spots in the oocyte nuclei of early egg chambers after Rad21^TEV^-myc cleavage, arguing against premature chromatid separation. This conclusion is also supported by our FISH results where Rad21^TEV^-myc cleavage was not observed to cause increased missegregation during the meiotic divisions. In contrast, increased numbers of centromere signals and increased missegregation were clearly detected in *ord* and *solo* mutants, which lack proteins specifically required for meiotic chromatid cohesion [40], [41], [48].

We consider the explanation that the normal meiotic chromosome segregation observed after TEV protease-mediated premature cleavage of Rad21^TEV^-myc might be due to putative residual non-cleaved Rad21^TEV^-myc to be highly unlikely. The same experimental strategy has proven to be extremely efficient in case of mitosis [44]. When Rad21^TEV^-myc is the sole Rad21 species in mitotically proliferating cells, ectopic Rad21^TEV^-myc cleavage results in a completely penetrant premature separation of sister chromatids in the first mitosis following TEV protease expression. It could be argued that a meiosis-specific factor might shield Rad21^TEV^-myc from TEV protease-mediated cleavage. However, in mouse oocytes, TEV protease-mediated inactivation of the meiotic α-kleisin was demonstrated to be efficient, arguing against a conserved shielding mechanism [37], [50]. In addition, we point out that TEV protease-mediated cleavage of Rad21^TEV^-myc before the meiotic division destroyed the maternal contribution of this mitotic α-kleisin so effectively that embryonic mitoses were completely defective.

Our hypothesis that Rad21 does not act as an essential meiotic α-kleisin during Drosophila female meiosis not only rests on the evidence obtained by TEV protease-mediated premature Rad21^TEV^-myc cleavage, but also on the unperturbed meiotic chromosome segregation observed after expression of Rad21^NC^-myc, in which the separase consensus cleavage sites were destroyed by site-directed mutations. An assay for the direct biochemical analysis of Rad21 cleavage by separase is still lacking in the Drosophila system. Therefore, it remains to be shown whether Rad21^NC^-myc is indeed resistant to separase-dependent proteolysis. However, the consequences resulting from expression of this variant in mitotically proliferating cells are perfectly consistent with the presence of separase-resistant cohesin rings that cannot be opened at the metaphase-to-anaphase transition in mitosis to liberate, and allow segregation of, the replicated sister chromatids. After expression during oogenesis, Rad21^NC^-myc is incorporated into meiotic chromatin and it is present in amounts sufficient to inhibit early embryonic mitoses. Thus, if separase-dependent removal of Rad21-containing cohesin was a crucial step during meiotic chromosome segregation, severe phenotypic consequences should not be restricted to early embryonic mitoses. Meiotic chromosome segregation would be predicted to be affected as well. However, we did not observe any meiotic abnormalities like chromosome bridges or missegregation of the X-chromosome in the FISH analyses.

Proper SC assembly depends on all three known meiotic α-kleisins in *C. elegans* and on both Rad21L and Rec8 in mouse spermatocytes [16], [21], [51]. Also in yeasts, which express only one meiotic α-kleisin, Rec8, SC integrity depends on Rec8 [11], [52]. However, no role for the mitotic α-kleisin Rad21/Scc1 in maintaining SC integrity has been found so far. Thus, our observation that the SC disassembles prematurely upon Rad21^TEV^-myc cleavage in Drosophila, demonstrates for the first time the dependence of SC maintenance on intact, Rad21-containing cohesin. However, this premature SC disassembly does not result in chromosome missegregation later in meiosis. We assume that the premature SC disassembly induced in our experiments occurs not early enough to interfere with crossover formation. A normal presence of chiasmata might therefore explain the absence of chromosome segregation defects. While our results clearly demonstrate that Rad21 is required for maintenance of the SC, we did not see an effect on establishment of the SC, not even when we used *nos-GAL4* to drive TEV protease expression early in the germarium. We suspect that *nos*-driven inactivation of Rad21^TEV^-myc is not fast or complete enough to profoundly affect SC maintenance in germarial stages, and only after a certain lag period enough TEV protease has accumulated to cleave sufficient Rad21^TEV^-myc, which then triggers SC disassembly.

An intriguing result of our experiments is the premature disassembly of the SC even when Rad21^TEV^-myc is ectopically cleaved, or when Rad21-EGFP is degraded, in the presence of one *Rad21* wild type allele. Because SC disassembly follows wild type kinetics in *Rad21* heterozygous females in the absence of ectopic Rad21 inactivation, the observed early SC disassembly after ectopic inactivation is not due to reduced *Rad21* gene dosage. Also, because cleavage at different positions and ectopic degradation of Rad21-EGFP resulted in premature SC disassembly, a dominant negative effect of the Rad21 fragments on SC structure is highly unlikely. One possible explanation can be based on a model that more than one cohesin ring is required at each linkage position to tether the SC to the chromosome cores. If just one out of two (or more) interconnected cohesin rings is opened by TEV protease action, or Rad21-EGFP proteolysis, linkage at this point would be abrogated despite the presence of uncleaved Rad21 in interconnecting rings. In support of this model, interaction studies between cohesin subunits led to the proposal of a “handcuff model” postulating interconnected cohesin rings [53].

The interaction between C(2)M and Rad21, which this work has revealed, suggests a model how the SC might be linked to cohesin within the chromosome cores. We propose that a direct interaction between the α-kleisin proteins C(2)M and Rad21 may provide a structural framework within the SC. We point out that so far we have been unable to confirm this interaction, which we have detected by co-immunoprecipitation from embryonic extracts and *in vitro* translation reactions, also by co-immunoprecipitation from ovary extracts, presumably because of technical difficulties (expression levels, insolubility of the SC associated proteins). While our demonstration of the C(2)M-Rad21 interaction is, to our knowledge, the first published report of an association of different α-kleisins, a homodimerization of human Rad21 has been demonstrated using yeast two-hybrid assays and immunoprecipitation experiments [53]. The reported localization of C(2)M as an LE component of the SC is also consistent with a direct connection to cohesin, which localizes to the chromosome cores [3], [45], [54]. Electron microscopy (EM) studies have mapped the N-terminus of C(2)M to the inner edge of the LEs [54]. According to our data, we would also expect the C-terminus of Rad21 to localize to this region of the SC. Staining of chromosome squash preparations indeed revealed a clear co-localization of Rad21^TEV^-myc and C(3)G (Fig. 1D), very similar to what has been observed previously for SMC1 [45]. Within the resolution limits of light microscopy, however, we cannot address the question, where Rad21^TEV^-myc exactly localizes within the SC. Analysis of the SC in Rad21^TEV^-myc expressing flies via immuno-EM will help to resolve this issue. Our inability to detect uncleaved Rad21^TEV^-myc localizing to meiotic chromatin in whole mount preparations may be due to epitope masking by a component, which might have been lost during the extensive washing steps with detergent-containing buffer in the chromosome squash preparations.

Taken together, we put forward a model in which at least two types of cohesin complexes are required during Drosophila oogenesis. Firstly, cohesin connecting the chromosome cores to the components of the SC contains the α-kleisin Rad21, possibly composed of multiple interconnected rings. Secondly, cohesin complexes holding together sister chromatids, either contain a very loosely conserved α-kleisin, which awaits to be discovered, or one of the non-kleisin cohesion proteins SOLO or ORD. In the latter case, it will be interesting to find out whether these proteins are substrates of separase.

## Materials and Methods

### Drosophila stocks and transgene construction

Flies expressing variants of Rad21, which are TEV protease cleavable and C-terminally fused to ten copies of the human c-myc epitope tag, in a *Rad21* mutant background, have been described [44]. Expression of these variants is driven by the ubiquitously active α-tubulin 84B promoter. The *nanos-GAL4* driver line (y^1^ w*; P{w[+mC] = GAL4-nos.NGT}40), the maternal triple driver (*MTD-GAL4*) [55], as well as the *c(2)M^EP2115^* stock [3], [39] were obtained from the Drosophila stock center (Bloomington, Indiana). The maternal *alpha-tubulin GAL4* (*mat-GAL4*) driver line has been described previously [56]. As source for TEV protease, we constructed transgenes encoding a modified enzyme (NLS-V5-TEV^S219V^), which possesses an N-terminal nuclear localization signal (NLS) followed by a V5 epitope tag and a valine instead of a serine residue at position 219, resulting in inhibition of self-cleavage and in about twofold higher activity levels [57]. We have exclusively used NLS-V5-TEV^S219V^ in this study and, for simplicity, refer to it as TEV protease throughout. To allow TEV protease expression during oogenesis, the NLS-V5-TEV^S219V^ coding sequence was cloned into pUASP1 [58]. Transgenic strains were established after injection into *w^1^* embryos using established procedures.

To obtain flies expressing a C(2)M variant tagged at its C-terminus with six copies of the hemagglutinin tag (6×HA) under control of the *c(2)m* genomic regulatory sequences, we replaced the 10×myc tag in a progenitor plasmid of the construct pCaSpeR-*gC(2)M-myc* [39] by the coding sequence for 6×HA. Briefly, a BamHI-XbaI-fragment containing the 3′-terminal part of *c(2)m* including the 10×myc encoding sequence was subcloned into pBSSK+ (Stratagene). An AgeI site was introduced immediately upstream the *c(2)m* stop codon by inverse PCR using the oligonucleotides C(2)M7 (5′- GGTGAGACCGGTTGAATATTTTTAGATAATTTTTTTCAAG-3′) and C(2)M8 (5′-CGTTCAACCGGTCTCACTCAGCATAAGATTG-3′) to yield pBSSK^+^ - BamHI-*C(2)M*-(AgeI)-XbaI. This step also removed the sequence encoding 10×myc. Next, an XhoI-BamHI fragment containing the 5′-terminal region of *c(2)m* including flanking genomic sequences was cloned into pBSSK^+^-BamHI-*C(2)M*-(AgeI)-XbaI resulting in pBSSK^+^-*gC(2)M*-(AgeI). The sequence encoding the 6×HA tag was obtained from the plasmid pUASP-*HA-Sse* [59] and cloned into the unique AgeI site of pBSSK^+^-*gC(2)M*-(AgeI). Finally, the complete insert was transferred as a 4.2 kb NotI-Asp718 fragment into the pattB vector [60]. Transgenic lines were generated by germline transformation of pattB-*gC(2)M-HA* into *y^1^*, *w^1^*, *M[vas-int]ZH2A; M[3x3P-RFP,attP']ZH51D* embryos [60].

To obtain lines carrying a functional *Rad21-EGFP* transgene, a construct similar to *Rad21-10myc* was generated. Briefly, the *EGFP* coding sequence was amplified using the primers SH257 (5′-CGTCTGTTCGAAAACCCAAAAATTGGCGGCGGCATGGTGAGCAAGG-3′) and SH258 (5′-CGTCTGTTCGAACTACTTGTACAGCTCGTCCATGC-3′) and cloned into the naturally occurring BstBI-site upstream of the *Rad21* translational stop codon in the *Rad21* cDNA clone LD14219 (BDGP). After introducing an additional Acc65I site in the polylinker upstream of the *Rad21* coding sequence, the complete *Rad21*-*EGFP* fragment was cloned as an Acc65I fragment into the modified pCaSpeR vector used for generating *Rad21^TEV^-myc* lines [44]. This vector allows expression of genes inserted in the unique Acc65I site under control of the ubiquitous active α-tubulin 84B promoter. Transgenic lines were established after P-element mediated germ-line transformation using pCaSpeR{*w^+^, αtub-Rad21-EGFP*} and injection into embryos derived from parents with the genotype *Rad21^ex3^/TM3, Ser*.

For the construction of a putative separase-resistant variant of Rad21, Rad21^NC^-myc, we employed a PCR-based strategy to exchange the codons 175 and 474 specifying arginines within the separase consensus sequences EXXR into codons specifying alanine residues. We have chosen these two sites because they align well with the known separase cleavage sites in Scc1/Rad21 from humans and yeasts [23]. As template for the PCR reactions, the Rad21 cDNA-based plasmid clone *pUAS-Rad21-10myc* [44] was used. A first 625 bp fragment comprising the Rad21 5′-UTR up to the region encoding the mutated first separase consensus cleavage site (EIIA) was PCR-amplified using the primers SH341 (ATAAGGCCGGCCACGAGACAGTTTTAGGTGATG) and SH342 (GAAGGTATACTGCAGGCTATAATTTCAGGCGTTTCTGC). A second 910 bp fragment corresponding to the Rad21 region from the mutated first putative separase cleavage site (EIIA) up to the mutated second separase consensus cleavage site (EVLA) was PCR-amplified using the primers SH343 (TTATAGCCTGCAGTATACCTTCAAATATTAATGATAAAA) and SH344 (TTCGCAGCTAGCACTTCCGGAGCTTCCAAACT). A third 1208 bp fragment corresponding to the Rad21 region from the mutated second separase consensus cleavage site (EVLA), through the C-terminal fused c-myc tag, was PCR-amplified using the primers SH345 (GGAAGTGCTAGCTGCGAATCATAAATCTCTAGGG) and SH346 (GTAGGCGCGCCATTAAAACAGATTTACATTCAACTT). The three PCR-generated DNA fragments partially overlap in the regions encoding the mutated separase consensus cleavage sites. After purification using the PCR purification kit (Thermo Scientific), the three PCR products were pooled and served as template for a final PCR using the flanking primers SH341 and SH346. The final 2696 bp PCR-product was cloned as an FseI/AscI fragment into a modified pUASP-vector containing unique FseI and AscI sites within its multiple cloning site. Transgenic lines were generated by P-element mediated germline transformation of *w^1^*-embryos using established procedures. For expression of this Rad21 variant in the developing eye, the *ey-GAL4* [61] driver line was used and for expression during oogenesis the *MTD-GAL4* driver line [55].

For the construction of the transgene *P*{*w^+^, UASP-NSlmb-vhh-GFP4*}, the EcoRI – XbaI insert fragment was isolated from *P*{*w^+^, UAST-NSlmb-vhh-GFP4*} [46], and inserted into the corresponding sites of pUASP1 [58]. Transgenic lines were generated by P-element mediated germline transformation of *w^1^*-embryos using established procedures.

For deGradFP dependent destruction of Rad21-EGFP during oogenesis, we generated w*; P{*w^+^, UASP-NSlmb-vhh-GFP4*}*II.1*/*nos-GAL4; Rad21^ex3^, P*{*w^+^, αtub-Rad21-EGFP*} *III.1*/*Rad21^ex3^, P*{*w^+^, αtub-Rad21-EGFP*} *III.1* females by standard crossing schemes. As controls, we also generated w*; P{*w^+^, UASP-NSlmb-vhh-GFP4*}*II.1*/*nos-GAL4; Rad21^ex3^, P*{*w^+^, αtub-Rad21-EGFP*}*III.1*/+ as well as w*; P{*w^+^, UASP-NSlmb-vhh-GFP4*}*II.1*/+; *Rad21^ex3^, P*{*w^+^, αtub-Rad21-EGFP*}*III.1*/*Rad21^ex3^, P*{*w^+^, αtub-Rad21-EGFP*}*III.1* females.

### Immunoblotting and immunoprecipitation experiments

For the immunoblotting experiments shown in Fig. 1, ovaries of 4–5 day old females fattened with yeast were dissected in 1× PBS and stage 14 oocytes were isolated and homogenized in SDS gel sample buffer. Protein samples were separated on Tris-glycine based polyacrylamide gels and blotted onto nitrocellulose membranes. For detection of myc epitope tags, HA epitope tags, V5 epitope tags, FLAG epitope tags and α-tubulin, the mouse monoclonal antibodies 9E10 [62], 12CA5 [63], anti-V5 (Invitrogen), anti-FLAG (Sigma) and DM1A (Sigma) were used, respectively. A guinea pig polyclonal antibody against Rad21 [39] and a rabbit antibody against EGFP [64] have been described. For detection of bound antibodies on immunoblots, the horseradish peroxidase based system from p.j.k was used according to the manufacturer's recommendations.

For the immunoprecipitation experiments shown in Fig. 4A, embryos were collected on apple juice agar plates for 1.5 h at 25°C. After dechorionization, the eggs were homogenized in 4× volume of lysis buffer (50 mM HEPES at pH 7.5, 60 mM NaCl, 3 mM MgCl_2_, 1 mM CaCl_2_, 0.2% Triton X-100, 0.2% Nonidet NP-40, 10% glycerol, 2 mM Pefabloc, 2 mM Benzamidin, 10 µg/ml Aprotinin, 2 µg/ml Pepstatin A, 10 µg/ml Leupeptin). The extracts were centrifuged and the supernatants were used for immunoprecipitation with anti-HA agarose beads (Roche). After 3 hours incubation at 4°C under rotation, the beads were washed 5× with lysis buffer and transferred into mobicol columns (MoBiTec). Bound proteins were eluted by adding 3× SDS sample buffer (6% SDS, 0.3 M β-mercaptoethanol, 30% glycerol, 0.3% bromophenol blue, 0.15 M Tris/HCl, pH 6.8) and boiling of the sample. The immunoprecipitates as well as samples of the input fractions and supernatants after precipitation were analyzed by immunoblotting.

For the *in vitro* interaction assays, proteins were synthesized using the TNT SP6 coupled reticulocyte lysate system (Promega) allowing coupled *in vitro* transcription and translation. To obtain the coding region of the SC components (C(3)G, C(2)M, Corona) and the cohesion proteins (SMC1, ORD, SOLO), RNA from ovaries was isolated and cDNA was synthesized using RevertAid H Minus M-MuLV Reverse Transcriptase (Fermentas) according to the manufacturer's recommendations. As templates for the TNT reactions, the reading frames of the respective genes were cloned into the expression vector pCS2 (F/A) [64] or derivatives thereof, allowing an N-terminal translational fusion with three copies of the FLAG epitope tag or with six copies of the myc epitope tag. For co-expression, equal amounts of plasmid constructs were added to the components of the TNT kit. Synthesized proteins were labeled by incorporation of [^35^S]methionine. For immunoprecipitation, anti-Flag or anti-myc agarose beads (Sigma) were used.

### Immunofluorescence microscopy

Ovaries were dissected in 1× PBS and fixed at room temperature for 20 min in a mixture of 300 µl heptane and 150 µl ovary fixation solution (1× PBS, 0.5% Nonidet NP 40 and 2% para-formaldehyde). Fixed ovaries were blocked for 1 h in PBS containing 0.2% Tween (PBTw) and 10% normal goat serum (NGS). Spread preparations of chromosomes were done as previously described [45]. Rabbit antibodies against Cenp-C [65] and against C(3)G [66] have been described and were used at a 1∶3,000 dilution. For some experiments, we used an anti C(3)G antibody we have raised in guinea pigs by immunization with a bacterially expressed C(3)G fragment corresponding to the C-terminus (aa 565–743). A rat antibody against Cid/Cenp-A (4F8, [67]) was diluted 1∶200. For SMC1 staining, a polyclonal antiserum was raised in rabbits using a bacterially expressed protein fragment corresponding to the N-terminal 133 amino acids of SMC1. The affinity purified antibody was used at a 1∶400 dilution. Antibodies against the HA epitope tag (Roche), the myc epitope tag (Sigma), and the V5 epitope tag (Invitrogen) were used at 1∶10, 1∶10 and 1∶500, respectively. All primary antibodies were diluted in PBTw +10% NGS. After washing twice in PBTw, secondary goat antibodies conjugated with Alexa 488 or Cy3 (Molecular Probes) were applied for 2 h in PBTw containing 5% NGS, followed by additional washes in PBTw. DNA was stained with Hoechst 33258 (1 µg/ml). Fluorescence images were acquired with a Leica SP5 confocal system (Leica Microsystems, Germany) or a Zeiss Axioplan 2 epifluorescence microscope. All images were processed using ImageJ v1.41 (National Institutes of Health, USA). For scoring SC disassembly, we have recorded the stages of those egg chambers showing complete SC disassembly, as indicated by the presence of only dot-like C(3)G signals within the oocyte chromatin and strong C(3)G staining of the oocyte nucleoplasm. The assignment of the stages was done based on size of the egg chambers as determined by equatorial focal planes. Because these planes only rarely allowed illustration of the oocyte nuclei, non-equatorial, and consequently smaller, sections containing the oocyte nuclei are shown in figs. 1, 2, 3, S5 and S7. To assess the significance of the differences in SC disassembly timing (Fig. 3A), we tested pairwise between the respective control situations (no TEV or no NSlmb-vhh-GFP4 expression) and the Rad21 degradation situations (TEV or NSlmb-vhh-GFP4 expression in *Rad21^TEV^-myc*, *Rad21^ex^* or *Rad21-EGFP*, *Rad21^ex^* background, respectively) using the Mann-Whitney U test (STATISTICA, StatSoft, Tulsa, OK, USA).

### Fluorescent *in situ* hybridization (FISH)

The X chromosome-specific 359 bp repeat was amplified by PCR with Drosophila genomic DNA as template [68]. The PCR product was digested overnight with a mixture of the restriction enzymes AluI, HaeIII, Tru1I, MspI, RsaI, and Sau3AI. Digested DNA was precipitated, dissolved in water, denatured at 100°C for 1 min and chilled on ice. The AATAT repeat specific for chromosome 4 was synthesized as a single-stranded oligonucleotide ((AATAT)_6_; Metabion international AG, Germany). 3′-Tailing of the single stranded DNAs with the reactive nucleotide Aminoallyl dUTP analog was done by using Terminal deoxynucleotidyl Transferase (Roche) at 37°C for 2 h in a reaction mixture containing 200 mM Na-Cacodylate (pH 7.2), 100 µM DTT, 1 mM CoCl_2_, 50 µM Aminoallyl dUTP (ARES DNA Alexa Fluor 555/647 labeling kit, Molecular probes) and 5 µM unlabeled dTTP. Reactions were stopped by adding 5 mM EDTA. Aminoallyl-conjugated probes were precipitated, dissolved in water and labeled with Alexa Fluor 555 or Alexa Fluor 647 in labeling buffer for 2 h in the dark, followed by quenching of the reactions with 150 µM hydroxylamine. Labeled probes were precipitated and dissolved in elution buffer.

FISH was done on stage 14 oocytes as described in [69] with some modifications. Oocytes were fixed in heptane/oocyte fixation solution, rinsed three times in 2× SSCT (0.3 M NaCl, 30 mM sodium citrate, 0.1% Tween 20), sequentially washed with 2× SSCT-20% formamide, 2× SSCT-40% formamide, and 2× SSCT-50% formamide for 10 min each followed by incubation in fresh 2× SSCT-50% formamide for 1–2 hrs at 37°C. The oocytes were transferred to 36 µl of hybridization buffer (20% dextrane sulfate, 15% formamide in 2× SSCT) and 100 ng of each fluorescently labelled probe was added. Probe and chromosomal DNA were denatured at 91°C for 2 min and the hybridization reaction was carried out overnight at 37°C. After hybridization, pre-warmed (37°C) 2× SSCT-50% formamide was added to the sample. Oocytes were washed three times with pre-warmed 2× SSCT-50% formamide, once with 2× SSCT-40% formamide, and 2× SSCT-20% formamide for 10 min/wash. Then, the oocytes were washed three times with 2× SSCT for 10 min each, rinsed three times with PBST and treated with Hoechst 33258 (1 µg/ml in PBS) to stain DNA. Finally, the oocytes were washed once with PBS for 5 min and mounted in 70% glycerol, 50 mM Tris-Cl (pH 9.5), 10 mg/ml propyl gallate, 0.5 mg/ml p-phenylenediamine in 1× PBS.

To enrich for oocytes progressing through meiosis II, approximately 300 females fattened for three days on yeast were put in collection cages and after a pre-collection for 1 h at 25°C, eggs were collected every 20 to 40 minutes for 5 hours. The eggs were immediately dechorionized and fixed with Methanol. Eggs from all collections were pooled and subjected to FISH as described above.

## Supporting Information

Figure S1 Expression profile of TEV-protease during early stages of oogenesis. The anterior part of ovarioles are shown. The different stages of development are given above the panels. Within the germaria, regions 2a, 2b, and 3 are designated r2a, r2b and r3, respectively. DNA was labelled with Hoechst 33258 and TEV protease was detected with anti-V5 antibodies directed against the V5-TEV protease fusion protein. In the merged panels, DNA is shown in red and V5-TEV protease in green. (A) TEV protease expression driven by *mat-GAL4*. (B) TEV protease expression driven by *nos-GAL4*. Scale bars are 5 µm.
(TIF)

Figure S2 The SC is established in germaria of females in which Rad21^TEV^-myc is ectopically cleaved. Immunofluorescence analysis of germaria from females with *GAL4*-driven expression of TEV protease in a *Rad21^TEV^-myc* rescue background (mat-Gal4/UAS-TEV; Rad21^ex^, Rad21^TEV^-myc/Rad21^ex^, Rad21^TEV^-myc, top row or nos-Gal4/UAS-TEV; Rad21^ex^, Rad21^TEV^-myc/Rad21^ex^, Rad21^TEV^-myc, bottom row). DNA was stained with Hoechst 33258 and C(3)G was labeled with anti-C(3)G antibodies. In the left column, an overview of the germaria is presented and the selected cells are shown enlarged in the other panels. In the merged images, DNA is shown in red and the C(3)G-signal in green. 3–4 individual confocal z-sections are presented as maximum projections for the overview, and single sections for the individual enlarged nuclei. Scale bar is 5 µm.
(TIF)

Figure S3 Initiation of SC disassembly in germaria/early egg chambers after Rad21^TEV^-myc cleavage. Chromosome spread analysis of germaria from females with *nos-GAL4*-driven expression of TEV protease in a *Rad21^TEV^-myc* rescue background (genotype: *nos-GAL4/UAS-TEV; Rad21^ex^, Rad21^TEV^-myc/Rad21^ex^, Rad21^TEV^-myc*). The Rad21^TEV^-myc signals in C(3)G-positive cells appear more punctate and fuzzy when compared to the situation when no TEV protease is expressed (top row, compare with Fig. 1D), indicative of progressing Rad21^TEV^-myc cleavage. The two adjacent C(3)G-positive cells indicate that these cells derive from region 2a or region 2b of the germarium. The individual nucleus shown in the bottom row is likely derived from late region 3 or an early egg chamber. In this nucleus, Rad21^TEV^-myc staining is even less pronounced (fewer dot-like signals) and the C(3)G staining is less thread-like and fuzzier when compared with earlier stages. In the merged images, DNA is shown in blue, anti-myc in red and the C(3)G-signal in green. Scale bar is 5 µm.
(TIF)

Figure S4 Rad21-EGFP is degraded after *mat-GAL4* driven expression of *NSlmb-vhhGFP4*. Extracts were prepared from 3–8 h old embryos expressing *Rad21-EGFP* and *UAS-NSlmb-vhhGFP4* under control of *mat-GAL4* (+SCF deg.), or from control embryos not expressing *UAS-NSlmb-vhhGFP4* (−SCF deg.). Proteins were separated by SDS-PAGE, blotted, and the blot was probed with anti-EGFP, anti-Tubulin, and anti-Rad21 antibodies. The number of embryo equivalents loaded is given on top of each lane.
(TIF)

Figure S5 Remnants of the SC after forced Rad21^TEV^-myc cleavage co-localize with centromeres. Ovarioles from females with the genotype *mat-GAL4/UAS-TEV; Rad21^ex^, Rad21^TEV^-myc/Rad21^ex^, Rad21^TEV^-myc* were fixed and labelled with antibodies against C(3)G and the centromere marker Cid/Cenp-A. In the images on the left an overview of the selected region of the respective ovariole is shown. In the merged panels is DNA in red, C(3)G in green and Cid/Cenp-A in blue. Scale bars are 5 µm.
(TIF)

Figure S6 Rad21^TEV^-myc cleavage results in massive defects during mitotic divisions in early embryos. 0–60 min old embryos derived from mothers not expressing TEV protease with the genotype *mat-GAL4/CyO; Rad21^ex^, Rad21^TEV^-myc/Rad21^ex^, Rad21^TEV^-myc* (A) or from mothers expressing TEV protease with the genotype *mat-GAL4/UAS-TEV; Rad21^ex^, Rad21^TEV^-myc/Rad21^ex^, Rad21^TEV^-myc* (B–G) were fixed and labelled with antibodies against α-tubulin (tub) and a DNA stain (DNA). In the merged panels is DNA in red, and tubulin in green. Scale bar is 10 µm. (A) metaphase plates from a control embryo progressing through mitosis 11 in the syncytial blastoderm stage. (B) Compact and bright spindle indicative of a prolonged metaphase arrest. (C–G) scattered DNA masses organizing multiple and/or multipolar spindles.
(TIF)

Figure S7 Venus-SOLO localizes to centromeres after Rad21^TEV^-myc cleavage. Ovarioles from females with the genotype *nos-GAL4/UAS-Venus-SOLO* (top row; nos>Venus-SOLO) or *nos-GAL4/UAS-Venus-SOLO, UAS-TEV; Rad21^ex^, Rad21^TEV^-myc/Rad21^ex^, Rad21^TEV^-myc* (bottom rows; nos>Venus-SOLO after Rad21 cleavage) were fixed and labelled with antibodies against C(3)G (bottom row), EGFP (which recognizes Venus-SOLO; all rows) and the centromere marker Cid/Cenp-A (top two rows). In the images on the left, overviews are shown of the selected regions containing the oocyte nucleus within the respective ovarioles. In the merged panels is DNA in blue, C(3)G or Cid/Cenp-A in red and Venus-SOLO in green. Scale bars are 5 µm.
(TIF)

Figure S8 Phenotypic consequences of expression of Rad21^NC^-myc in the developing eye. Eyes of individuals with the genotype (A) *ey-GAL4/+; +/+* and (B, C) *ey-GAL4/+; UASP1-Rad21^NC^–myc III.15/+*. Flies were raised at 28°C.
(TIF)

## References

[1] Petronczki M, Siomos MF, Nasmyth K (2003) Un menage a quatre: the molecular biology of chromosome segregation in meiosis. Cell 112: 423–440. 12600308 10.1016/s0092-8674(03)00083-7

[2] McKim KS, Green-Marroquin BL, Sekelsky JJ, Chin G, Steinberg C, et al (1998) Meiotic synapsis in the absence of recombination. Science 279: 876–878. 9452390 10.1126/science.279.5352.876

[3] Manheim EA, McKim KS (2003) The Synaptonemal complex component C(2)M regulates meiotic crossing over in Drosophila. Curr Biol 13: 276–285. 12593793 10.1016/s0960-9822(03)00050-2

[4] Page SL, Hawley RS (2001) c(3)G encodes a Drosophila synaptonemal complex protein. Genes Dev 15: 3130–3143. 11731477 10.1101/gad.935001 PMC312841

[5] Oliveira RA, Nasmyth K (2010) Getting through anaphase: splitting the sisters and beyond. Biochem Soc Trans 38: 1639–1644. 21118140 10.1042/BST0381639

[6] Haering CH, Jessberger R (2012) Cohesin in determining chromosome architecture. Exp Cell Res 318: 1386–1393. 22472347 10.1016/j.yexcr.2012.03.016

[7] Guacci V, Koshland D, Strunnikov A (1997) A direct link between sister chromatid cohesion and chromosome condensation revealed through the analysis of MCD1 in S-cerevisiae. Cell 91: 47–57. 9335334 10.1016/s0092-8674(01)80008-8 PMC2670185

[8] Michaelis C, Ciosk R, Nasmyth K (1997) Cohesins: Chromosomal proteins that prevent premature separation of sister chromatids. Cell 91: 35–45. 9335333 10.1016/s0092-8674(01)80007-6

[9] Haering CH, Farcas AM, Arumugam P, Metson J, Nasmyth K (2008) The cohesin ring concatenates sister DNA molecules. Nature 454: 297–301. 18596691 10.1038/nature07098

[10] McNicoll F, Stevense M, Jessberger R (2013) Cohesin in gametogenesis. Curr Top Dev Biol 102: 1–34. 23287028 10.1016/B978-0-12-416024-8.00001-5

[11] Klein F, Mahr P, Galova M, Buonomo SB, Michaelis C, et al (1999) A central role for cohesins in sister chromatid cohesion, formation of axial elements, and recombination during yeast meiosis. Cell 98: 91–103. 10412984 10.1016/S0092-8674(00)80609-1

[12] Watanabe Y, Nurse P (1999) Cohesin Rec8 is required for reductional chromosome segregation at meiosis. Nature 400: 461–464. 10440376 10.1038/22774

[13] Gutierrez-Caballero C, Herran Y, Sanchez-Martin M, Suja JA, Barbero JL, et al (2011) Identification and molecular characterization of the mammalian alpha-kleisin RAD21L. Cell Cycle 10: 1477–1487. 21527826 10.4161/cc.10.9.15515

[14] Ishiguro K, Kim J, Fujiyama-Nakamura S, Kato S, Watanabe Y (2011) A new meiosis-specific cohesin complex implicated in the cohesin code for homologous pairing. EMBO Rep 12: 267–275. 21274006 10.1038/embor.2011.2 PMC3059921

[15] Lee J, Hirano T (2011) RAD21L, a novel cohesin subunit implicated in linking homologous chromosomes in mammalian meiosis. J Cell Biol 192: 263–276. 21242291 10.1083/jcb.201008005 PMC3172173

[16] Llano E, Herran Y, Garcia-Tunon I, Gutierrez-Caballero C, de Alava E, et al (2012) Meiotic cohesin complexes are essential for the formation of the axial element in mice. J Cell Biol 197: 877–885. 22711701 10.1083/jcb.201201100 PMC3384418

[17] Herran Y, Gutierrez-Caballero C, Sanchez-Martin M, Hernandez T, Viera A, et al (2011) The cohesin subunit RAD21L functions in meiotic synapsis and exhibits sexual dimorphism in fertility. EMBO J 30: 3091–3105. 21743440 10.1038/emboj.2011.222 PMC3160193

[18] Bannister LA, Reinholdt LG, Munroe RJ, Schimenti JC (2004) Positional cloning and characterization of mouse mei8, a disrupted allelle of the meiotic cohesin Rec8. Genesis 40: 184–194. 15515002 10.1002/gene.20085

[19] Revenkova E, Eijpe M, Heyting C, Hodges CA, Hunt PA, et al (2004) Cohesin SMC1 beta is required for meiotic chromosome dynamics, sister chromatid cohesion and DNA recombination. Nat Cell Biol 6: 555–562. 15146193 10.1038/ncb1135

[20] Xu H, Beasley MD, Warren WD, van der Horst GT, McKay MJ (2005) Absence of mouse REC8 cohesin promotes synapsis of sister chromatids in meiosis. Dev Cell 8: 949–961. 15935783 10.1016/j.devcel.2005.03.018

[21] Severson AF, Ling L, van Zuylen V, Meyer BJ (2009) The axial element protein HTP-3 promotes cohesin loading and meiotic axis assembly in C. elegans to implement the meiotic program of chromosome segregation. Genes Dev 23: 1763–1778. 19574299 10.1101/gad.1808809 PMC2720254

[22] Uhlmann F, Wernic D, Poupart MA, Koonin EV, Nasmyth K (2000) Cleavage of cohesin by the CD clan protease separin triggers anaphase in yeast. Cell 103: 375–386. 11081625 10.1016/s0092-8674(00)00130-6

[23] Hauf S, Waizenegger IC, Peters JM (2001) Cohesin cleavage by separase required for anaphase and cytokinesis in human cells. Science 293: 1320–1323. 11509732 10.1126/science.1061376

[24] Kudo NR, Anger M, Peters AH, Stemmann O, Theussl HC, et al (2009) Role of cleavage by separase of the Rec8 kleisin subunit of cohesin during mammalian meiosis I. J Cell Sci 122: 2686–2698. 19625504 10.1242/jcs.035287 PMC2909317

[25] Kudo NR, Wassmann K, Anger M, Schuh M, Wirth KG, et al (2006) Resolution of chiasmata in oocytes requires separase-mediated proteolysis. Cell 126: 135–146. 16839882 10.1016/j.cell.2006.05.033

[26] Buonomo SB, Clyne RK, Fuchs J, Loidl J, Uhlmann F, et al (2000) Disjunction of homologous chromosomes in meiosis I depends on proteolytic cleavage of the meiotic cohesin Rec8 by separin. Cell 103: 387–398. 11081626 10.1016/s0092-8674(00)00131-8

[27] Kitajima TS, Miyazaki Y, Yamamoto M, Watanabe Y (2003) Rec8 cleavage by separase is required for meiotic nuclear divisions in fission yeast. EMBO J 22: 5643–5653. 14532136 10.1093/emboj/cdg527 PMC213781

[28] Kitajima TS, Sakuno T, Ishiguro K, Iemura S, Natsume T, et al (2006) Shugoshin collaborates with protein phosphatase 2A to protect cohesin. Nature 441: 46–52. 16541025 10.1038/nature04663

[29] Riedel CG, Katis VL, Katou Y, Mori S, Itoh T, et al (2006) Protein phosphatase 2A protects centromeric sister chromatid cohesion during meiosis I. Nature 441: 53–61. 16541024 10.1038/nature04664

[30] Lee J, Yokota T, Yamashita M (2002) Analyses of mRNA expression patterns of cohesin subunits Rad21 and Rec8 in mice: germ cell-specific expression of rec8 mRNA in both male and female mice. Zoolog Sci 19: 539–544. 12130806 10.2108/zsj.19.539

[31] Parra MT, Viera A, Gomez R, Page J, Benavente R, et al (2004) Involvement of the cohesin Rad21 and SCP3 in monopolar attachment of sister kinetochores during mouse meiosis I. J Cell Sci 117: 1221–1234. 14970259 10.1242/jcs.00947

[32] Xu H, Beasley M, Verschoor S, Inselman A, Handel MA, et al (2004) A new role for the mitotic RAD21/SCC1 cohesin in meiotic chromosome cohesion and segregation in the mouse. EMBO Rep 5: 378–384. 15031719 10.1038/sj.embor.7400121 PMC1299032

[33] Prieto I, Tease C, Pezzi N, Buesa JM, Ortega S, et al (2004) Cohesin component dynamics during meiotic prophase I in mammalian oocytes. Chromosome Res 12: 197–213. 15125634 10.1023/b:chro.0000021945.83198.0e

[34] Gomez R, Valdeolmillos A, Parra MT, Viera A, Carreiro C, et al (2007) Mammalian SGO2 appears at the inner centromere domain and redistributes depending on tension across centromeres during meiosis II and mitosis. EMBO Rep 8: 173–180. 17205076 10.1038/sj.embor.7400877 PMC1796771

[35] Eijpe M, Offenberg H, Jessberger R, Revenkova E, Heyting C (2003) Meiotic cohesin REC8 marks the axial elements of rat synaptonemal complexes before cohesins SMC1beta and SMC3. J Cell Biol 160: 657–670. 12615909 10.1083/jcb.200212080 PMC2173354

[36] Prieto I, Pezzi N, Buesa JM, Kremer L, Barthelemy I, et al (2002) STAG2 and Rad21 mammalian mitotic cohesins are implicated in meiosis. EMBO Rep 3: 543–550. 12034751 10.1093/embo-reports/kvf108 PMC1084142

[37] Tachibana-Konwalski K, Godwin J, van der Weyden L, Champion L, Kudo NR, et al (2010) Rec8-containing cohesin maintains bivalents without turnover during the growing phase of mouse oocytes. Genes Dev 24: 2505–2516. 20971813 10.1101/gad.605910 PMC2975927

[38] Schleiffer A, Kaitna S, Maurer-Stroh S, Glotzer M, Nasmyth K, et al (2003) Kleisins: a superfamily of bacterial and eukaryotic SMC protein partners. Mol Cell 11: 571–575. 12667442 10.1016/s1097-2765(03)00108-4

[39] Heidmann D, Horn S, Heidmann S, Schleiffer A, Nasmyth K, et al (2004) The Drosophila meiotic kleisin C(2)M functions before the meiotic divisions. Chromosoma 113: 177–187. 15375666 10.1007/s00412-004-0305-5

[40] Bickel SE, Wyman DW, Orr-Weaver TL (1997) Mutational analysis of the Drosophila sister-chromatid cohesion protein ORD and its role in the maintenance of centromeric cohesion. Genetics 146: 1319–1331. 9258677 10.1093/genetics/146.4.1319 PMC1208078

[41] Yan R, McKee BD (2013) The cohesion protein SOLO associates with SMC1 and is required for synapsis, recombination, homolog bias and cohesion and pairing of centromeres in Drosophila Meiosis. PLoS Genet 9: e1003637. 23874232 10.1371/journal.pgen.1003637 PMC3715423

[42] Yan R, Thomas SE, Tsai JH, Yamada Y, McKee BD (2010) SOLO: a meiotic protein required for centromere cohesion, coorientation, and SMC1 localization in Drosophila melanogaster. J Cell Biol 188: 335–349. 20142422 10.1083/jcb.200904040 PMC2819681

[43] Webber HA, Howard L, Bickel SE (2004) The cohesion protein ORD is required for homologue bias during meiotic recombination. J Cell Biol 164: 819–829. 15007062 10.1083/jcb.200310077 PMC2172286

[44] Pauli A, Althoff F, Oliveira RA, Heidmann S, Schuldiner O, et al (2008) Cell-type-specific TEV protease cleavage reveals cohesin functions in Drosophila neurons. Dev Cell 14: 239–251. 18267092 10.1016/j.devcel.2007.12.009 PMC2258333

[45] Khetani RS, Bickel SE (2007) Regulation of meiotic cohesion and chromosome core morphogenesis during pachytene in Drosophila oocytes. J Cell Sci 120: 3123–3137. 17698920 10.1242/jcs.009977

[46] Caussinus E, Kanca O, Affolter M (2012) Fluorescent fusion protein knockout mediated by anti-GFP nanobody. Nat Struct Mol Biol 19: 117–121. 22157958 10.1038/nsmb.2180

[47] Takeo S, Lake CM, Morais-de-Sa E, Sunkel CE, Hawley RS (2011) Synaptonemal complex-dependent centromeric clustering and the initiation of synapsis in Drosophila oocytes. Curr Biol 21: 1845–1851. 22036182 10.1016/j.cub.2011.09.044

[48] Bickel SE, Orr-Weaver TL, Balicky EM (2002) The sister-chromatid cohesion protein ORD is required for chiasma maintenance in Drosophila oocytes. Curr Biol 12: 925–929. 12062057 10.1016/s0960-9822(02)00846-1

[49] Howard-Till RA, Lukaszewicz A, Novatchkova M, Loidl J (2013) A single cohesin complex performs mitotic and meiotic functions in the protist tetrahymena. PLoS Genet 9: e1003418. 23555314 10.1371/journal.pgen.1003418 PMC3610610

[50] Tachibana-Konwalski K, Godwin J, Borsos M, Rattani A, Adams DJ, et al (2013) Spindle assembly checkpoint of oocytes depends on a kinetochore structure determined by cohesin in meiosis I. Curr Biol 23: 2534–2539. 24291092 10.1016/j.cub.2013.10.052 PMC3898714

[51] Murdoch B, Owen N, Stevense M, Smith H, Nagaoka S, et al (2013) Altered cohesin gene dosage affects Mammalian meiotic chromosome structure and behavior. PLoS Genet 9: e1003241. 23408896 10.1371/journal.pgen.1003241 PMC3567145

[52] Molnar M, Bahler J, Sipiczki M, Kohli J (1995) The rec8 gene of Schizosaccharomyces pombe is involved in linear element formation, chromosome pairing and sister-chromatid cohesion during meiosis. Genetics 141: 61–73. 8536990 10.1093/genetics/141.1.61 PMC1206740

[53] Zhang N, Kuznetsov SG, Sharan SK, Li K, Rao PH, et al (2008) A handcuff model for the cohesin complex. J Cell Biol 183: 1019–1031. 19075111 10.1083/jcb.200801157 PMC2600748

[54] Anderson LK, Royer SM, Page SL, McKim KS, Lai A, et al (2005) Juxtaposition of C(2)M and the transverse filament protein C(3)G within the central region of Drosophila synaptonemal complex. Proc Natl Acad Sci U S A 102: 4482–4487. 15767569 10.1073/pnas.0500172102 PMC555515

[55] Petrella LN, Smith-Leiker T, Cooley L (2007) The Ovhts polyprotein is cleaved to produce fusome and ring canal proteins required for Drosophila oogenesis. Development 134: 703–712. 17215303 10.1242/dev.02766

[56] Micklem DR, Dasgupta R, Elliott H, Gergely F, Davidson C, et al (1997) The mago nashi gene is required for the polarisation of the oocyte and the formation of perpendicular axes in Drosophila. Curr Biol 7: 468–478. 9210377 10.1016/s0960-9822(06)00218-1

[57] Kapust RB, Tozser J, Fox JD, Anderson DE, Cherry S, et al (2001) Tobacco etch virus protease: mechanism of autolysis and rational design of stable mutants with wild-type catalytic proficiency. Protein Eng 14: 993–1000. 11809930 10.1093/protein/14.12.993

[58] Jäger H, Rauch M, Heidmann S (2005) The Drosophila melanogaster condensin subunit Cap-G interacts with the centromere-specific histone H3 variant CID. Chromosoma 113: 350–361. 15592865 10.1007/s00412-004-0322-4

[59] Jäger H, Herzig A, Lehner CF, Heidmann S (2001) Drosophila Separase is required for sister chromatid separation and binds to PIM and THR. Genes Dev 15: 2572–2584. 11581162 10.1101/gad.207301 PMC312799

[60] Bischof J, Maeda RK, Hediger M, Karch F, Basler K (2007) An optimized transgenesis system for Drosophila using germ-line-specific phiC31 integrases. Proc Natl Acad Sci U S A 104: 3312–3317. 17360644 10.1073/pnas.0611511104 PMC1805588

[61] Hazelett DJ, Bourouis M, Walldorf U, Treisman JE (1998) decapentaplegic and wingless are regulated by eyes absent and eyegone and interact to direct the pattern of retinal differentiation in the eye disc. Development 125: 3741–3751. 9716539 10.1242/dev.125.18.3741

[62] Evan GI, Lewis GK, Ramsay G, Bishop JM (1985) Isolation of monoclonal antibodies specific for human c-myc proto-oncogene product. Mol Cell Biol 5: 3610–3616. 3915782 10.1128/mcb.5.12.3610-3616.1985 PMC369192

[63] Niman HL, Houghten RA, Walker LE, Reisfeld RA, Wilson IA, et al (1983) Generation of protein-reactive antibodies by short peptides is an event of high frequency: implications for the structural basis of immune recognition. Proc Natl Acad Sci USA 80: 4949–4953. 6192445 10.1073/pnas.80.16.4949 PMC384165

[64] Herzog S, Nagarkar Jaiswal S, Urban E, Riemer A, Fischer S, et al (2013) Functional dissection of the Drosophila melanogaster condensin subunit Cap-G reveals its exclusive association with condensin I. PLoS Genet 9: e1003463. 23637630 10.1371/journal.pgen.1003463 PMC3630105

[65] Heeger S, Leismann O, Schittenhelm R, Schraidt O, Heidmann S, et al (2005) Genetic interactions of separase regulatory subunits reveal the diverged Drosophila Cenp-C homolog. Genes Dev 19: 2041–2053. 16140985 10.1101/gad.347805 PMC1199574

[66] Hong A, Lee-Kong S, Iida T, Sugimura I, Lilly MA (2003) The p27cip/kip ortholog dacapo maintains the Drosophila oocyte in prophase of meiosis I. Development 130: 1235–1242. 12588841 10.1242/dev.00352

[67] Padeken J, Mendiburo MJ, Chlamydas S, Schwarz HJ, Kremmer E, et al (2013) The nucleoplasmin homolog NLP mediates centromere clustering and anchoring to the nucleolus. Mol Cell 50: 236–249. 23562326 10.1016/j.molcel.2013.03.002

[68] Hsieh T, Brutlag D (1979) Sequence and sequence variation within the 1.688 g/cm3 satellite DNA of Drosophila melanogaster. J Mol Biol 135: 465–481. 231676 10.1016/0022-2836(79)90447-9

[69] Dernburg AF, Broman KW, Fung JC, Marshall WF, Philips J, et al (1996) Perturbation of nuclear architecture by long-distance chromosome interactions. Cell 85: 745–759. 8646782 10.1016/s0092-8674(00)81240-4

