# Supplementary figures and images for: The Cohesin Subunit Rad21 Is Required for Synaptonemal Complex Maintenance, but Not Sister Chromatid Cohesion, during Drosophila Female Meiosis
Source: PLoS Genet. 2014 Aug 7;10(8):e1004540. doi: 10.1371/journal.pgen.1004540 (PMC4125089; doi:10.1371/journal.pgen.1004540)

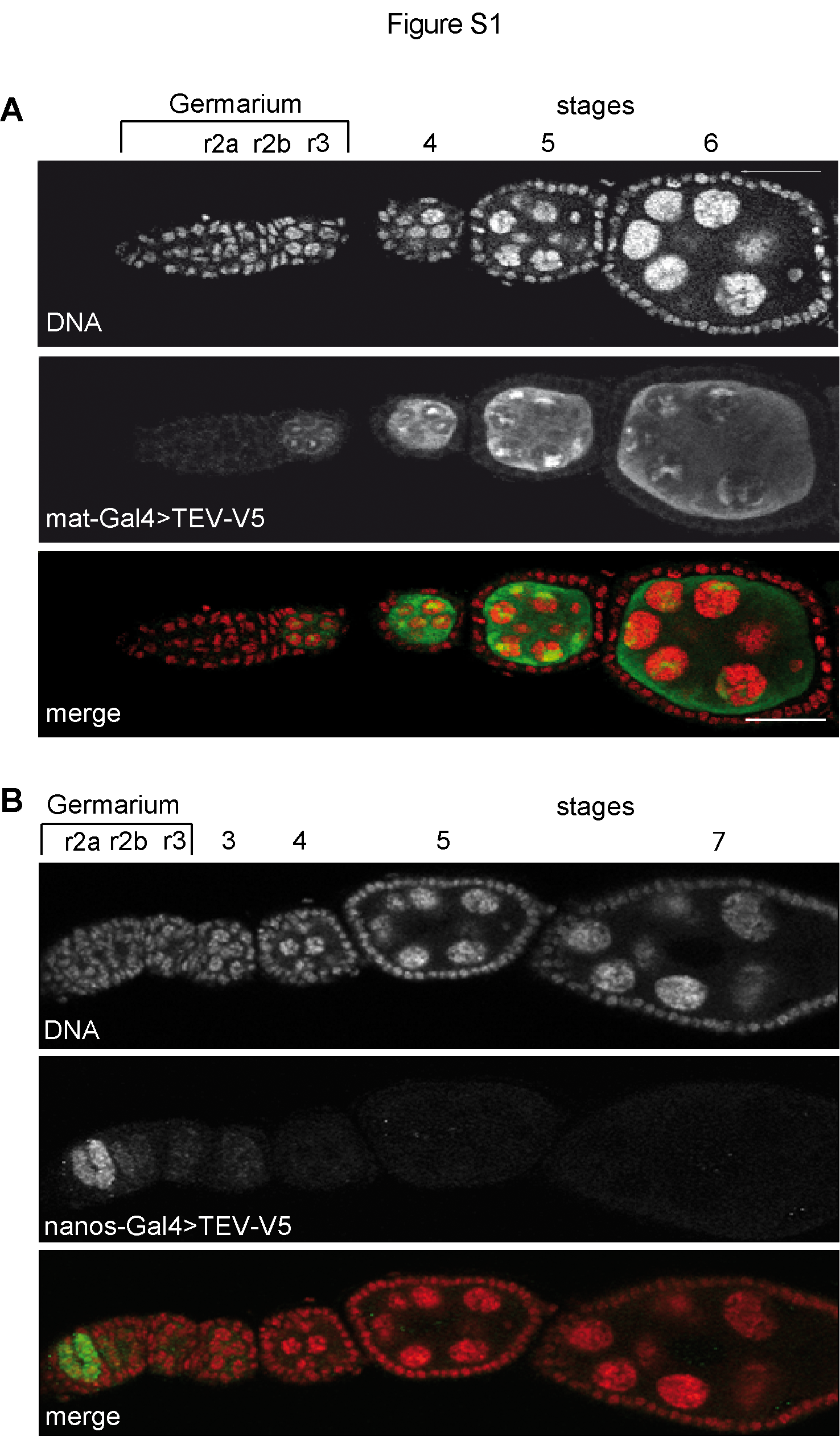

Supplement: Figure S1 — Expression profile of TEV-protease during early stages of oogenesis. The anterior part of ovarioles are shown. The different stages of development are given above the panels. Within the germaria, regions 2a, 2b, and 3 are designated r2a, r2b and r3, respectively. DNA was labelled with Hoechst 33258 and TEV protease was detected with anti-V5 antibodies directed against the V5-TEV protease fusion protein. In the merged panels, DNA is shown in red and V5-TEV protease in green. (A) TEV protease expression driven by mat-GAL4. (B) TEV protease expression driven by nos-GAL4. Scale bars are 5 µm. (TIF) [file pgen.1004540.s001.tif]

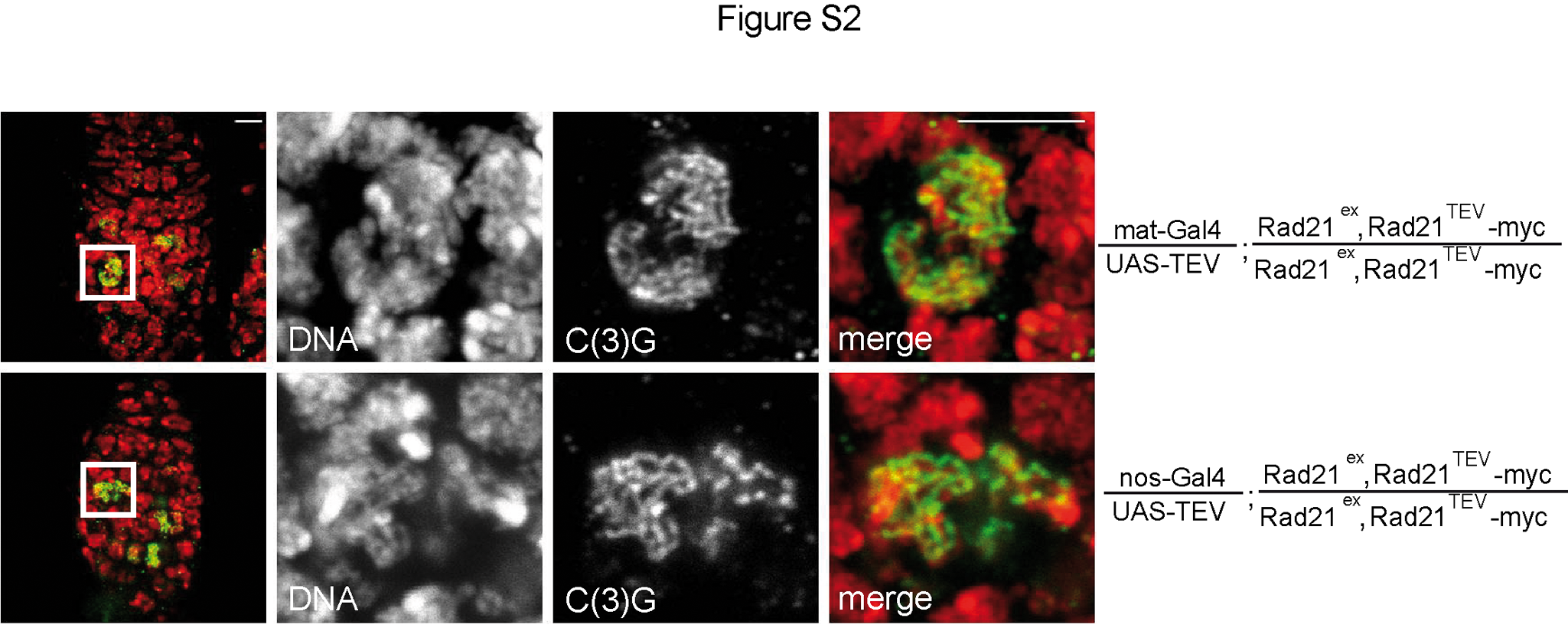

Supplement: Figure S2 — The SC is established in germaria of females in which Rad21TEV-myc is ectopically cleaved. Immunofluorescence analysis of germaria from females with GAL4-driven expression of TEV protease in a Rad21TEV-myc rescue background (mat-Gal4/UAS-TEV; Rad21ex, Rad21TEV-myc/Rad21ex, Rad21TEV-myc, top row or nos-Gal4/UAS-TEV; Rad21ex, Rad21TEV-myc/Rad21ex, Rad21TEV-myc, bottom row). DNA was stained with Hoechst 33258 and C(3)G was labeled with anti-C(3)G antibodies. In the left column, an overview of the germaria is presented and the selected cells are shown enlarged in the other panels. In the merged images, DNA is shown in red and the C(3)G-signal in green. 3–4 individual confocal z-sections are presented as maximum projections for the overview, and single sections for the individual enlarged nuclei. Scale bar is 5 µm. (TIF) [file pgen.1004540.s002.tif]

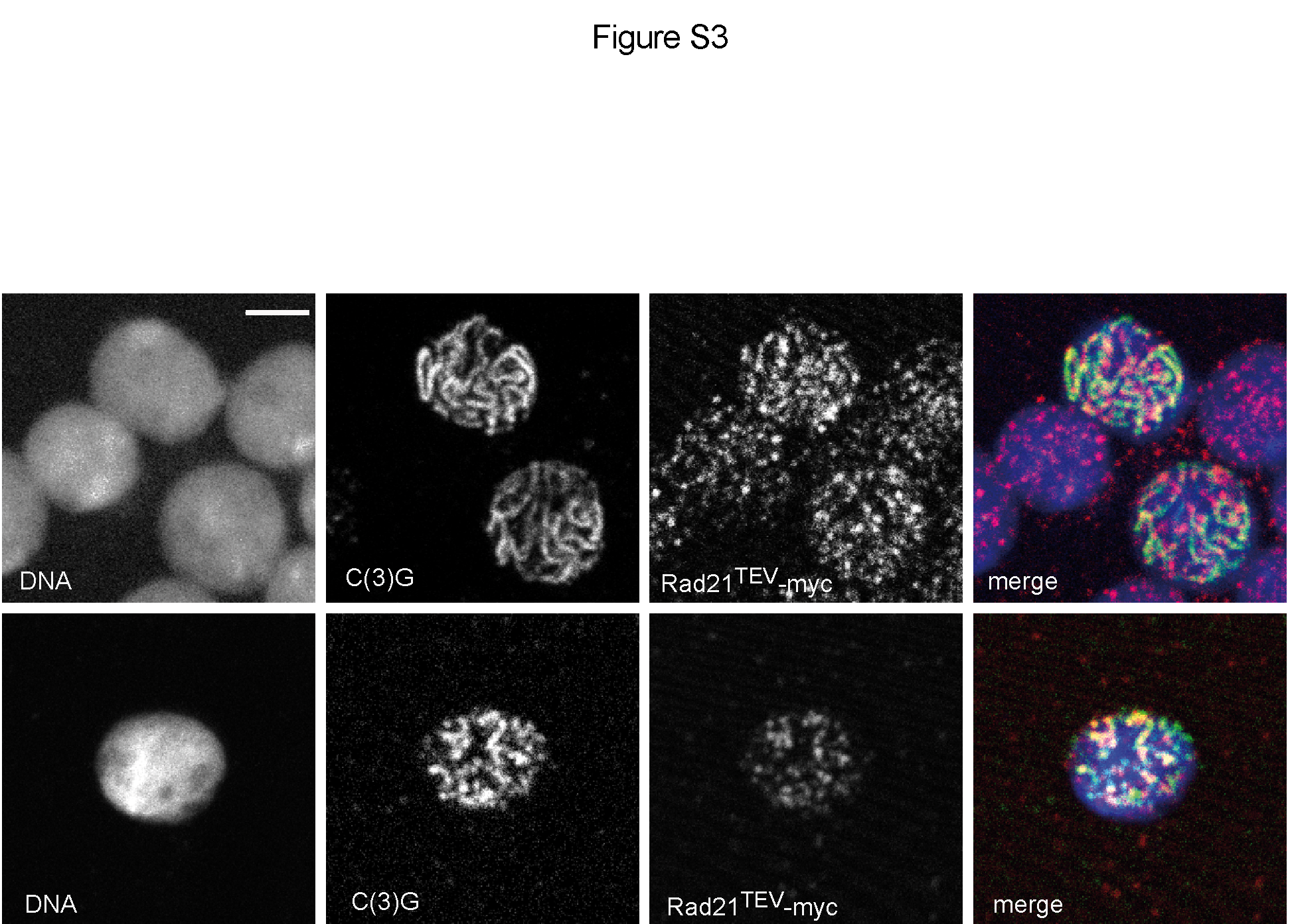

Supplement: Figure S3 — Initiation of SC disassembly in germaria/early egg chambers after Rad21TEV-myc cleavage. Chromosome spread analysis of germaria from females with nos-GAL4-driven expression of TEV protease in a Rad21TEV-myc rescue background (genotype: nos-GAL4/UAS-TEV; Rad21ex, Rad21TEV-myc/Rad21ex, Rad21TEV-myc). The Rad21TEV-myc signals in C(3)G-positive cells appear more punctate and fuzzy when compared to the situation when no TEV protease is expressed (top row, compare with Fig. 1D), indicative of progressing Rad21TEV-myc cleavage. The two adjacent C(3)G-positive cells indicate that these cells derive from region 2a or region 2b of the germarium. The individual nucleus shown in the bottom row is likely derived from late region 3 or an early egg chamber. In this nucleus, Rad21TEV-myc staining is even less pronounced (fewer dot-like signals) and the C(3)G staining is less thread-like and fuzzier when compared with earlier stages. In the merged images, DNA is shown in blue, anti-myc in red and the C(3)G-signal in green. Scale bar is 5 µm. (TIF) [file pgen.1004540.s003.tif]

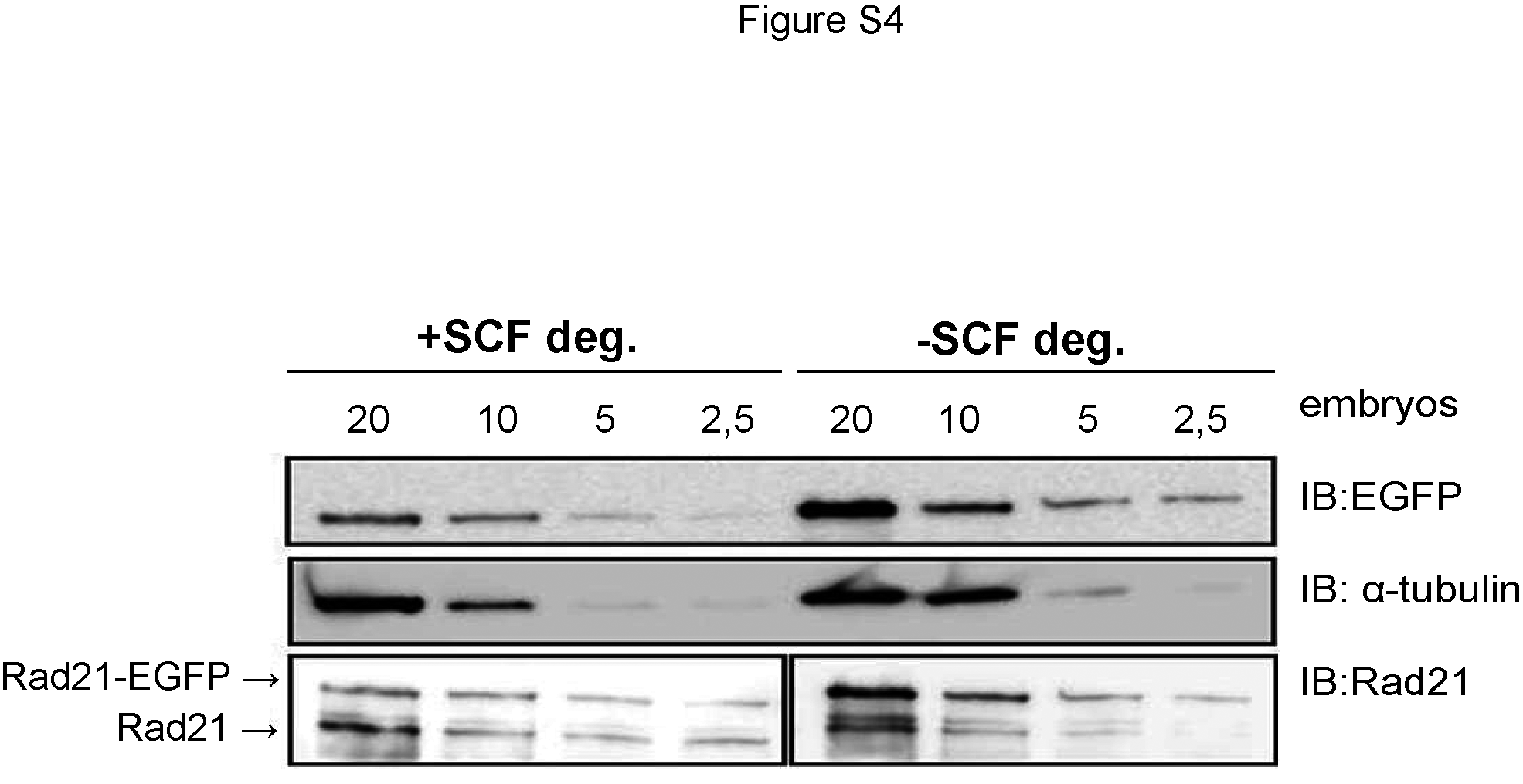

Supplement: Figure S4 — Rad21-EGFP is degraded after mat-GAL4 driven expression of NSlmb-vhhGFP4. Extracts were prepared from 3–8 h old embryos expressing Rad21-EGFP and UAS-NSlmb-vhhGFP4 under control of mat-GAL4 (+SCF deg.), or from control embryos not expressing UAS-NSlmb-vhhGFP4 (−SCF deg.). Proteins were separated by SDS-PAGE, blotted, and the blot was probed with anti-EGFP, anti-Tubulin, and anti-Rad21 antibodies. The number of embryo equivalents loaded is given on top of each lane. (TIF) [file pgen.1004540.s004.tif]

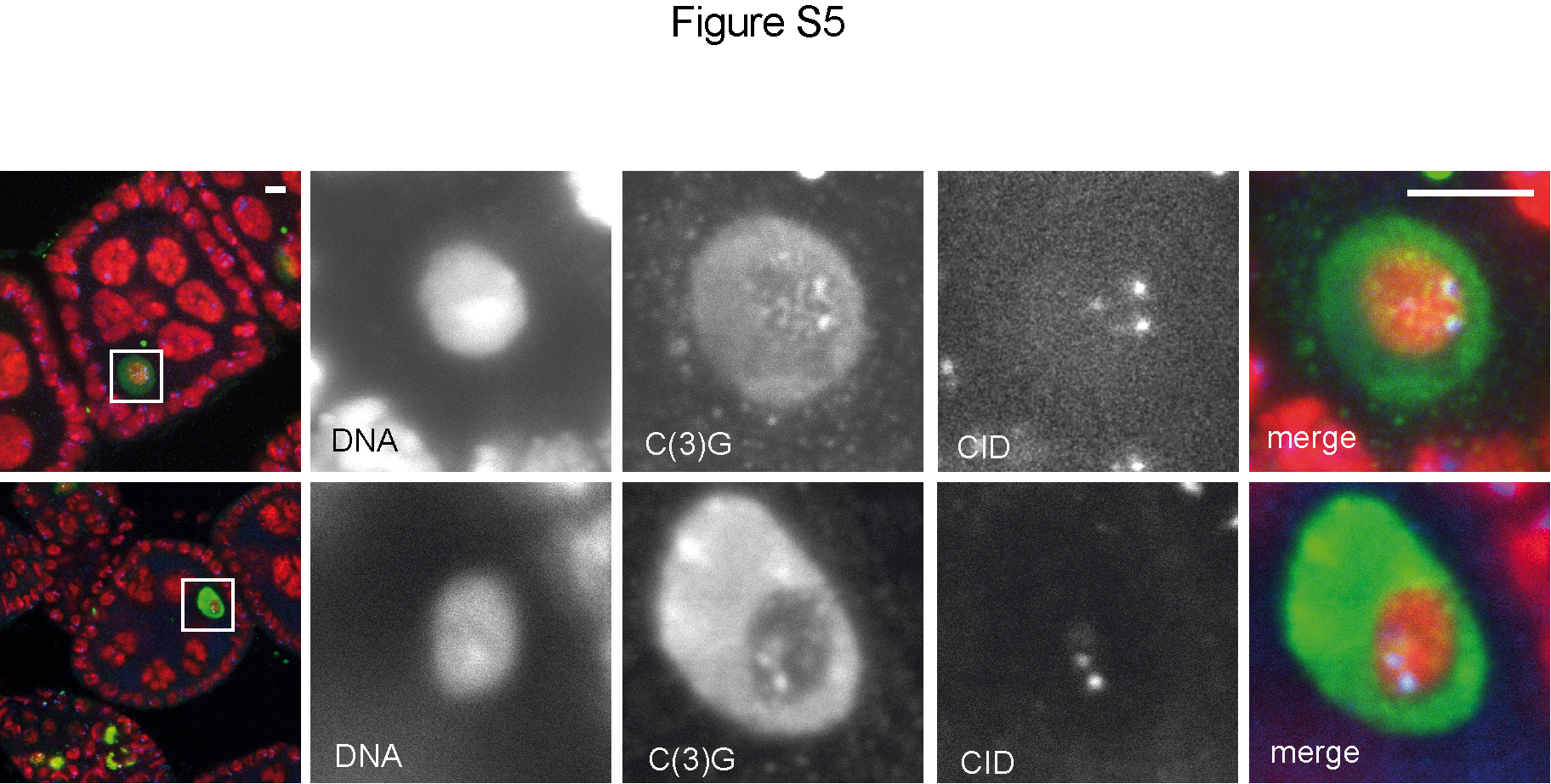

Supplement: Figure S5 — Remnants of the SC after forced Rad21TEV-myc cleavage co-localize with centromeres. Ovarioles from females with the genotype mat-GAL4/UAS-TEV; Rad21ex, Rad21TEV-myc/Rad21ex, Rad21TEV-myc were fixed and labelled with antibodies against C(3)G and the centromere marker Cid/Cenp-A. In the images on the left an overview of the selected region of the respective ovariole is shown. In the merged panels is DNA in red, C(3)G in green and Cid/Cenp-A in blue. Scale bars are 5 µm. (TIF) [file pgen.1004540.s005.tif]

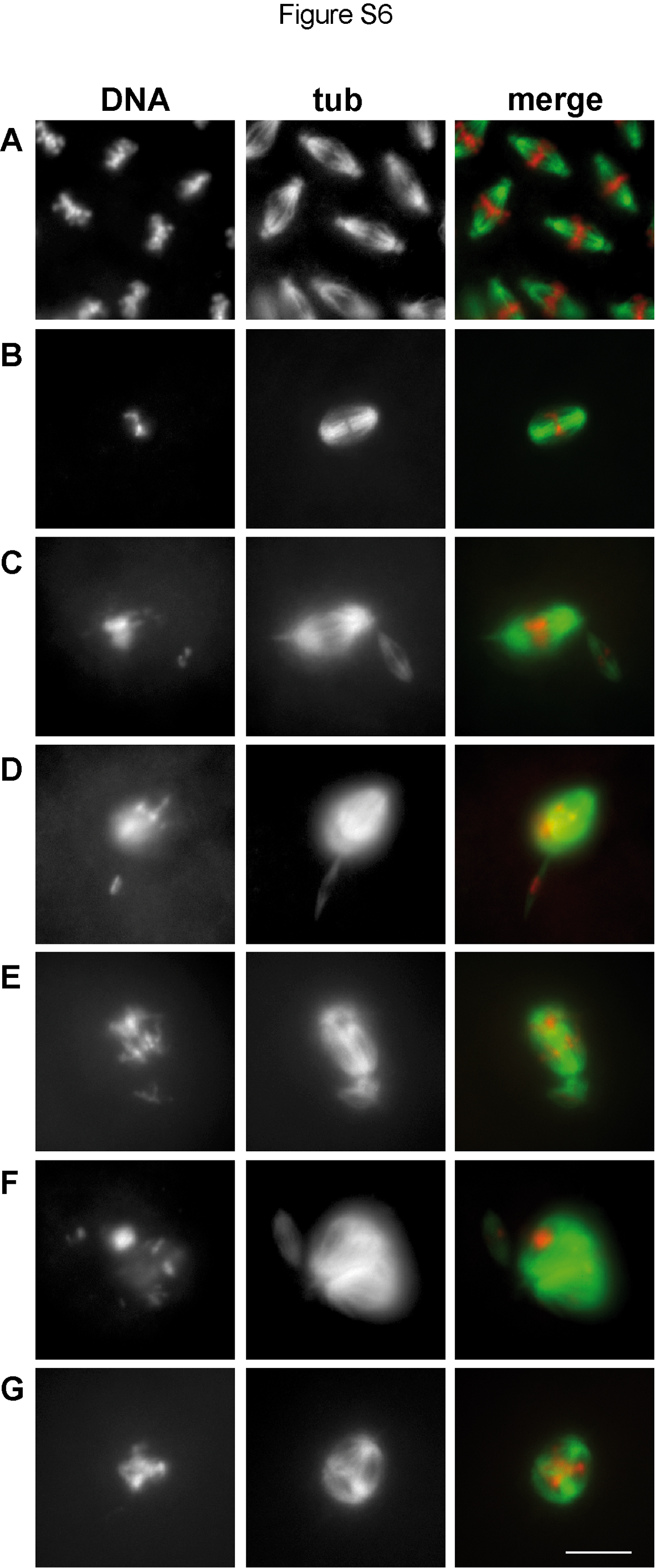

Supplement: Figure S6 — Rad21TEV-myc cleavage results in massive defects during mitotic divisions in early embryos. 0–60 min old embryos derived from mothers not expressing TEV protease with the genotype mat-GAL4/CyO; Rad21ex, Rad21TEV-myc/Rad21ex, Rad21TEV-myc (A) or from mothers expressing TEV protease with the genotype mat-GAL4/UAS-TEV; Rad21ex, Rad21TEV-myc/Rad21ex, Rad21TEV-myc (B–G) were fixed and labelled with antibodies against α-tubulin (tub) and a DNA stain (DNA). In the merged panels is DNA in red, and tubulin in green. Scale bar is 10 µm. (A) metaphase plates from a control embryo progressing through mitosis 11 in the syncytial blastoderm stage. (B) Compact and bright spindle indicative of a prolonged metaphase arrest. (C–G) scattered DNA masses organizing multiple and/or multipolar spindles. (TIF) [file pgen.1004540.s006.tif]

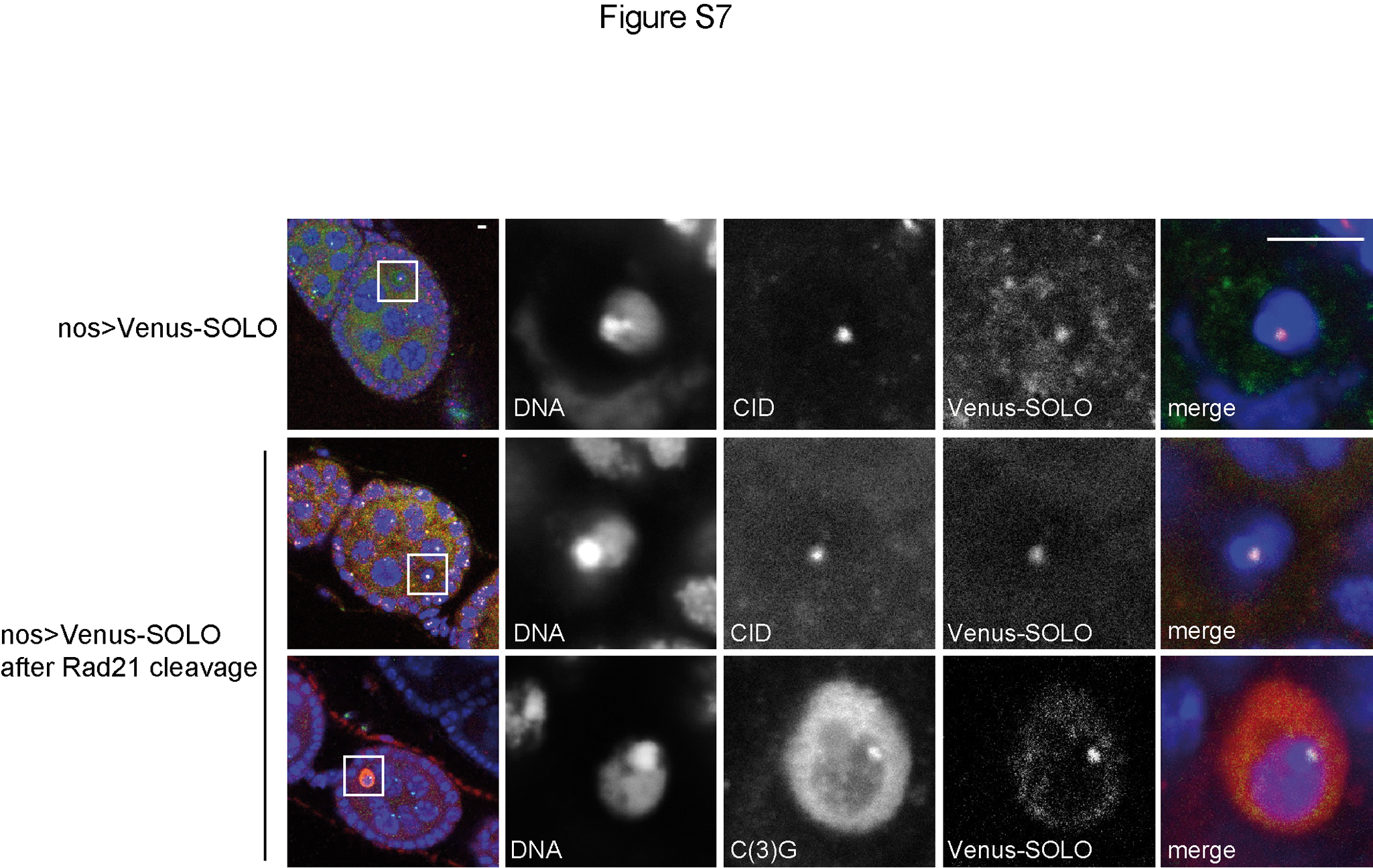

Supplement: Figure S7 — Venus-SOLO localizes to centromeres after Rad21TEV-myc cleavage. Ovarioles from females with the genotype nos-GAL4/UAS-Venus-SOLO (top row; nos>Venus-SOLO) or nos-GAL4/UAS-Venus-SOLO, UAS-TEV; Rad21ex, Rad21TEV-myc/Rad21ex, Rad21TEV-myc (bottom rows; nos>Venus-SOLO after Rad21 cleavage) were fixed and labelled with antibodies against C(3)G (bottom row), EGFP (which recognizes Venus-SOLO; all rows) and the centromere marker Cid/Cenp-A (top two rows). In the images on the left, overviews are shown of the selected regions containing the oocyte nucleus within the respective ovarioles. In the merged panels is DNA in blue, C(3)G or Cid/Cenp-A in red and Venus-SOLO in green. Scale bars are 5 µm. (TIF) [file pgen.1004540.s007.tif]

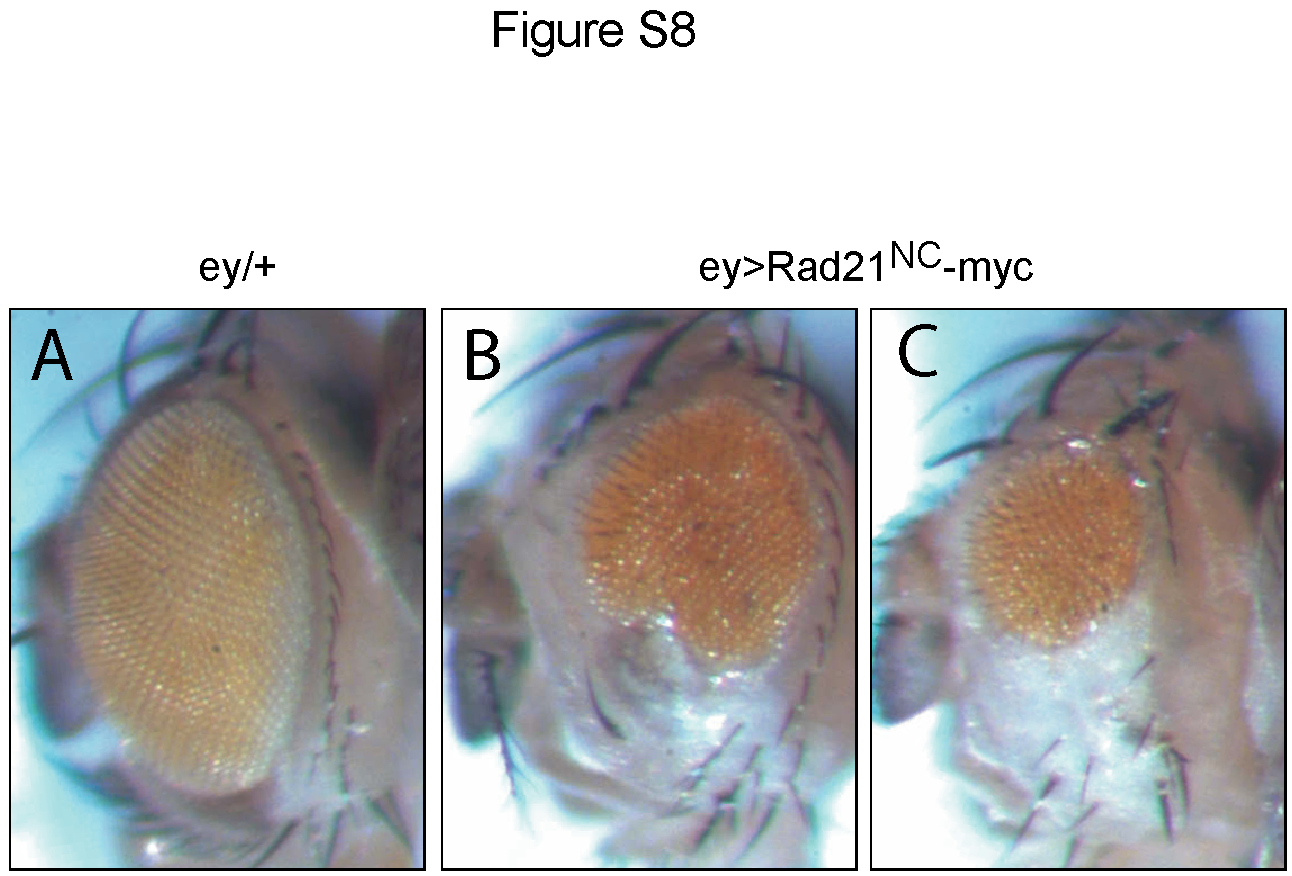

Supplement: Figure S8 — Phenotypic consequences of expression of Rad21NC-myc in the developing eye. Eyes of individuals with the genotype (A) ey-GAL4/+; +/+ and (B, C) ey-GAL4/+; UASP1-Rad21NC–myc III.15/+. Flies were raised at 28°C. (TIF) [file pgen.1004540.s008.tif]
